# Supplementary material for: Inhibition of the de novo pyrimidine biosynthesis pathway limits ribosomal RNA transcription causing nucleolar stress in glioblastoma cells
Source: PLoS Genet. 2020 Nov 17;16(11):e1009117. doi: 10.1371/journal.pgen.1009117 (PMC7707548; doi:10.1371/journal.pgen.1009117)
Supplement: S1 Fig — (A) mRNA expression of lower grade (II-III) to higher grade (IV/Glioblastoma) gliomas from patients whose tumors are archived in the CGGA database. (B) Table showing the characteristics of the cell lines used in the present manuscript. (C) Brequinar levels quantified by LC-MS/MS in LN229 cells treated with 0.1 μM brequinar. Numerical values for each of the experiments represented are available in S6 Data. (PDF) [file pgen.1009117.s001.pdf]

A

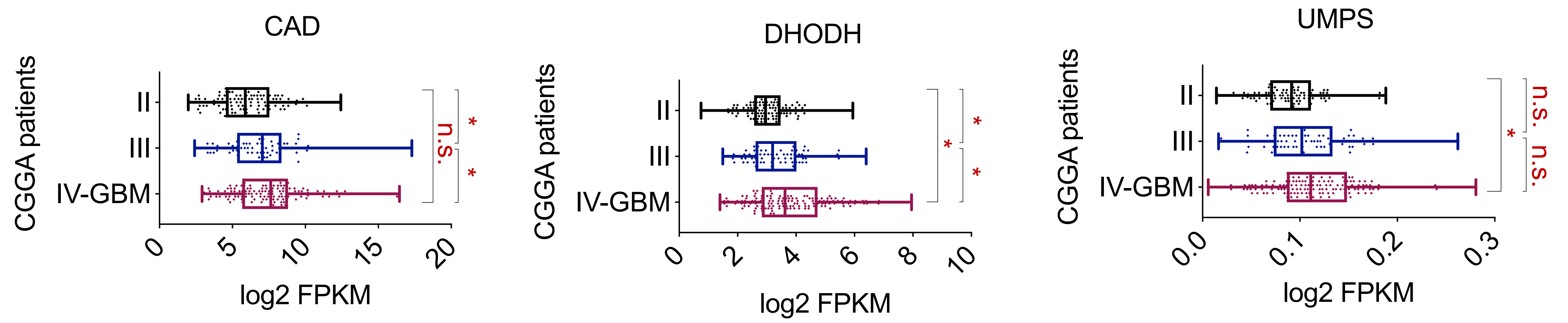

B

	origin	cell type	Gender	Age	p14ARF status	p53 status	p53 activity (p21 expression)
**Human astrocytes**	commercial	astrocytes	unknown	unknown	deleted (CRISPR)	WT	unknown
**ARPE-19**	commercial	retinal epithelial	Male	19	unknown	unknown	unknown
**LN229**	commercial	glioblastoma (WHO IV grade)	Female	60	deleted	P98L	active
**SF188**	commercial	glioblastoma (WHO IV grade)	Male	8	unknown	G266E	inactive
**GBM9**	patient-derived	glioblastoma (WHO IV grade)	unknown	unknown	unknown	unknown (non deleted)	inactive

C

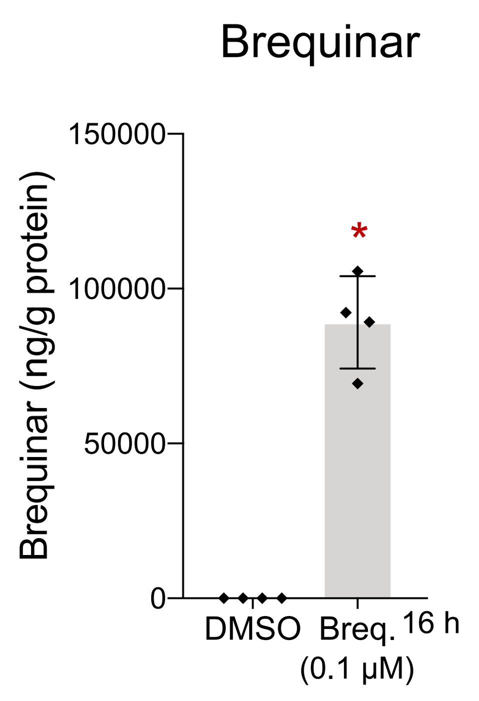

Figure S1
