# Supplementary figures and images for: Inhibition of the de novo pyrimidine biosynthesis pathway limits ribosomal RNA transcription causing nucleolar stress in glioblastoma cells
Source: PLoS Genet. 2020 Nov 17;16(11):e1009117. doi: 10.1371/journal.pgen.1009117 (PMC7707548; doi:10.1371/journal.pgen.1009117)

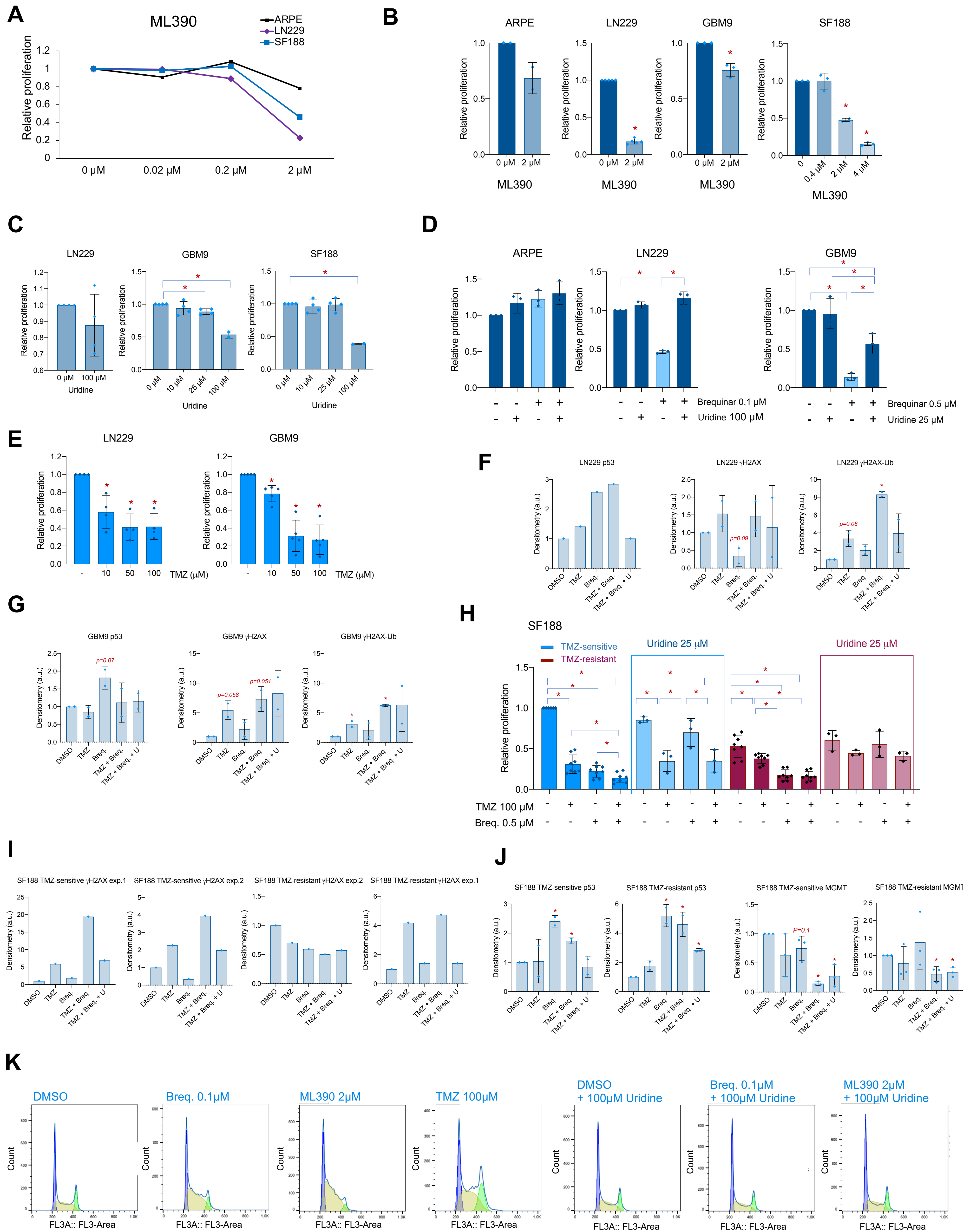

Figure S2

Supplement: S2 Fig — (A) Relative proliferation of non-transformed retinal epithelial ARPE and the glioblastoma cells LN229, GBM9 and SF188 in the presence of increasing amounts of the DHODH inhibitor, ML390. Media with drugs was replaced every 2 days for 6 days. Proliferation was assessed by crystal violet staining. (B) Relative proliferation of ARPE cells and the LN229, GBM9 and SF188 GBM cells in the presence of ML390. Media with drugs was replaced every 2 days for 6 days. N = 2–5. (C) Relative proliferation of LN229, GBM9 and SF188 cells in increasing amounts of uridine. Media with uridine was added the day after seeding. Media and uridine were replaced every 2 days, and proliferation was 6 days after treatment. N = 4. (D) Relative proliferation of ARPE, LN229 and GBM9 cells with or without brequinar and with or without uridine. Media with drugs and uridine were replaced the day after cell seeding, and proliferation assessed after 4 days. N = 3. (E) Relative proliferation of the LN229 and GBM9 cells in the presence of increasing amounts of temozolomide (TMZ). Media with drug were replaced the day after seeding, and proliferation assessed after 4 days. N = 4–5. (F) Western blot quantification by Image J of Fig 2F and additional experimental replicates. (G) Western blot quantification by Image J of Fig 2G and additional experimental replicates. N = 2. (H) Relative proliferation of SF188 TMZ-sensitive or -resistant cells with or without TMZ, brequinar or brequinar + TMZ with or without uridine normalize to SF188 TMZ-sensitive DMSO condition. Also see Fig 2J. (I) Western blot quantification by Image J of γH2AX in Fig 2K and additional experimental replicates. The results are represented for each experimental replicate. N = 2. (J) Western blot quantification by Image J of p53 and MGMT in Fig 2K and additional experimental replicates. N = 2 for p53, N = 3 for MGMT. (K) Cell cycle representation of results in Fig 2L. Asterisks indicate p-values ≦ 0.05. Numerical values for each of the expe [file pgen.1009117.s002.pdf]

**A**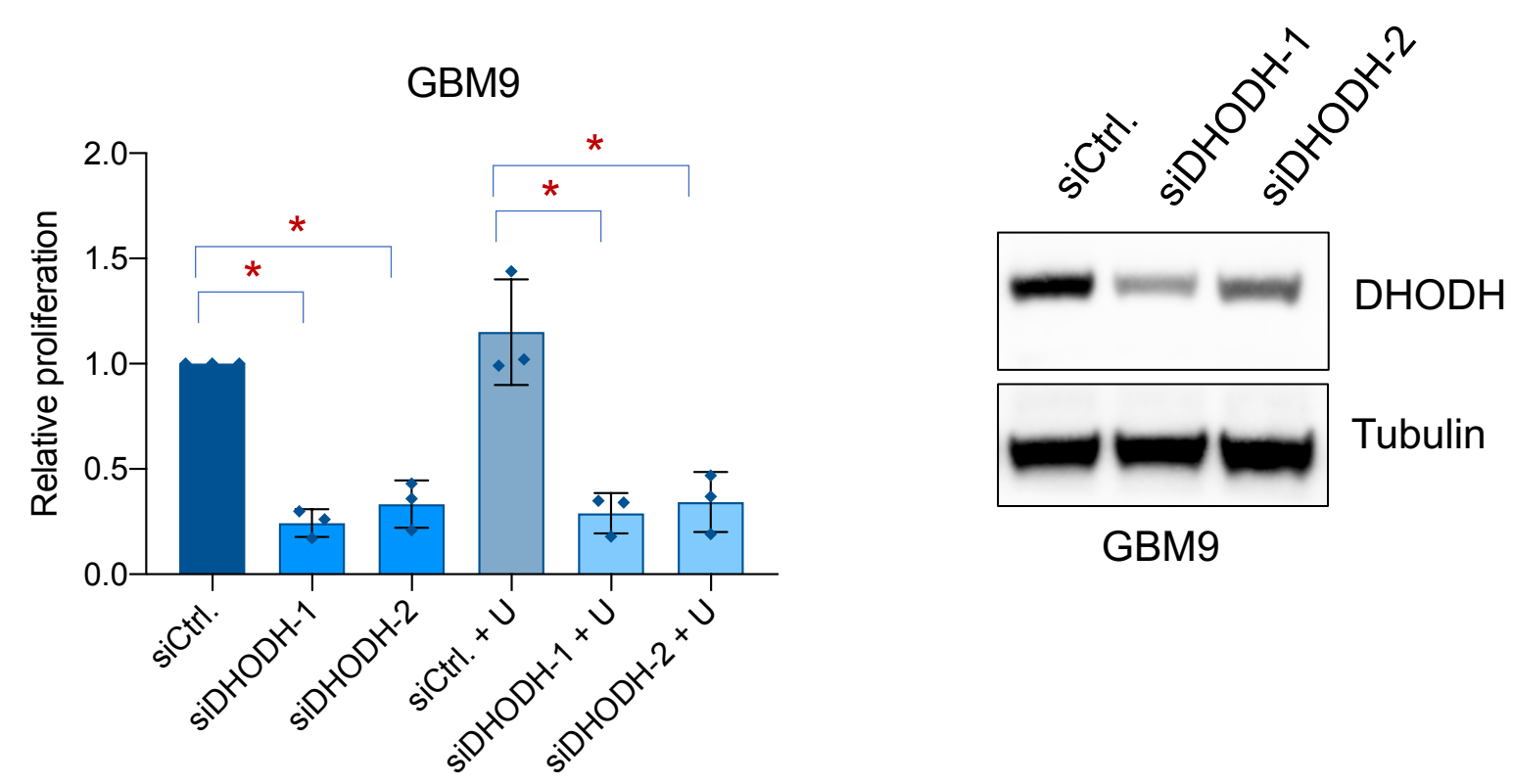**B**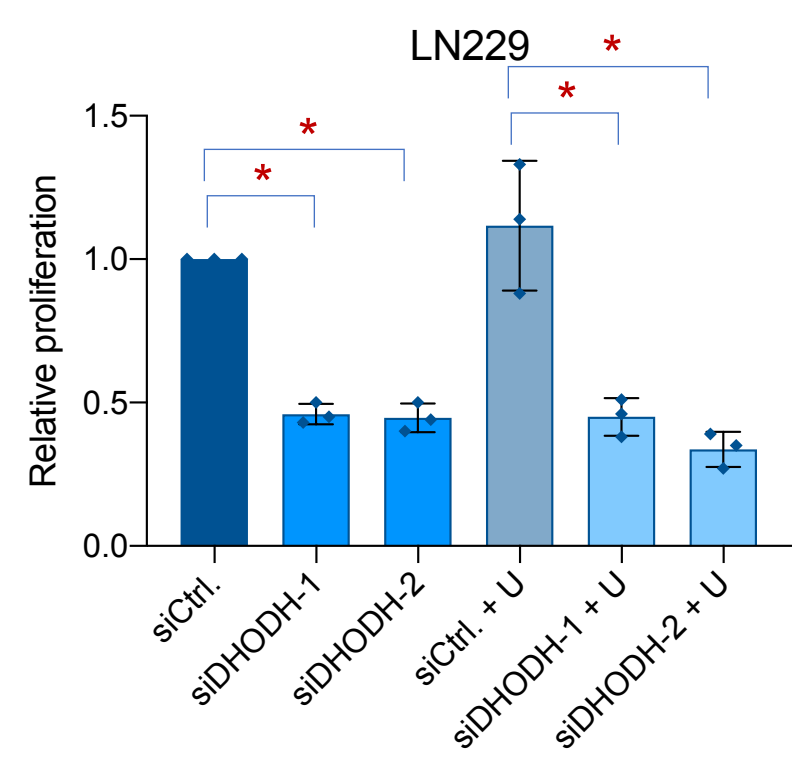**C**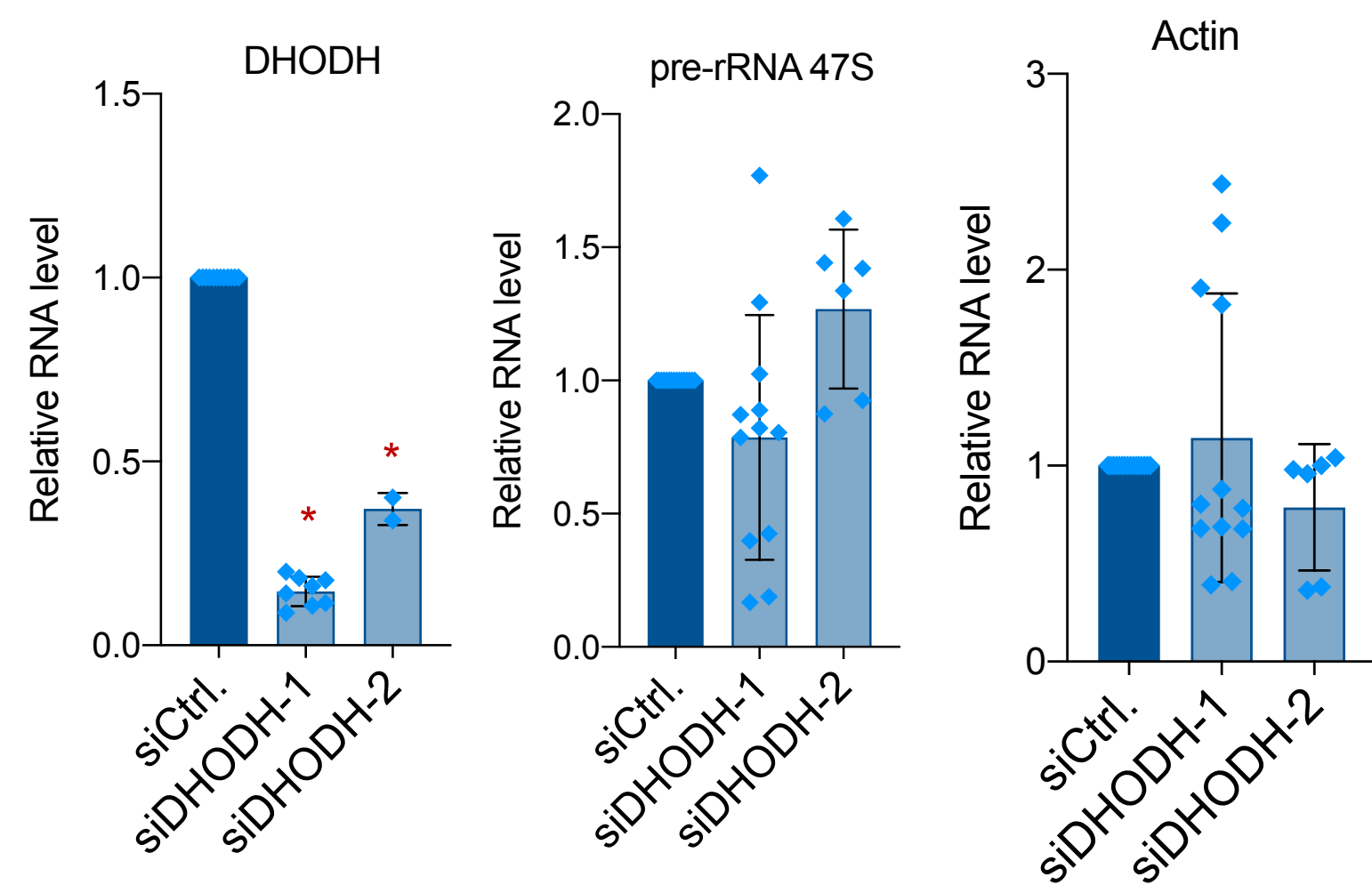**D**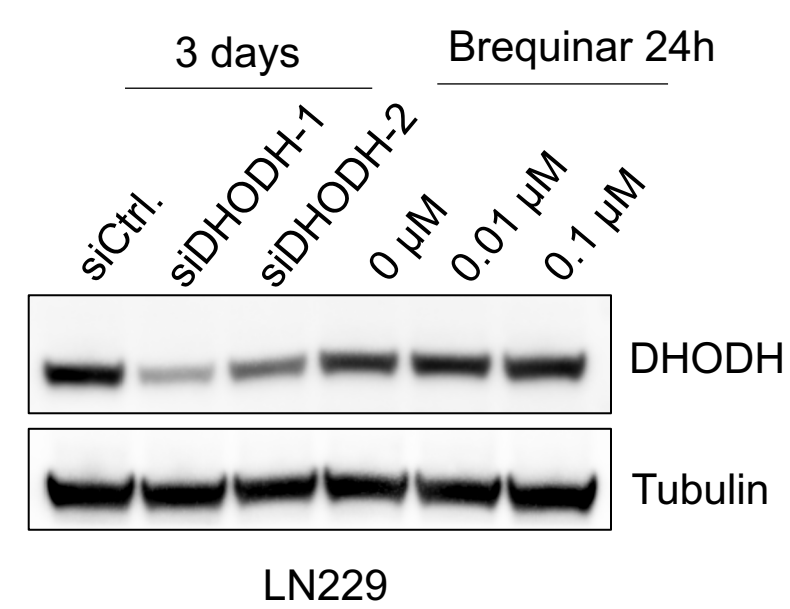**E**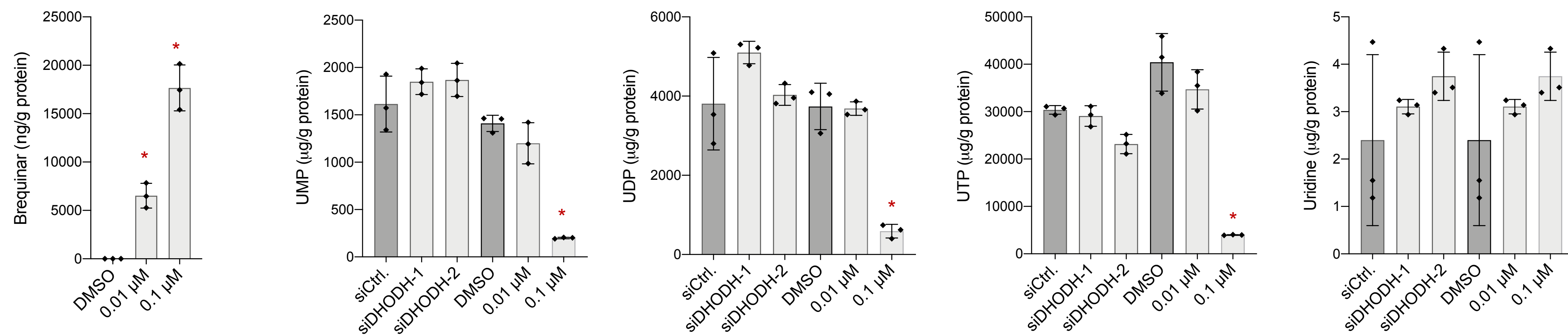

Figure S3

Supplement: S3 Fig — (A) Relative proliferation of GBM9 cells upon DHODH knockdown with or without uridine. Proliferation was assessed by crystal violet staining after 4 days of siRNA transfection. Fresh media with uridine were added the day after siRNA transfection. Uridine did not rescue the effects of DHODH knockdown in proliferation. Western blot for DHODH on the right panel. N = 3. (B) Relative proliferation of LN229 cells upon DHODH knockdown with or without uridine. Proliferation was assessed by crystal violet staining after 4 days of siRNA transfection. Fresh media with uridine were added the day after siRNA transfection. Uridine did not rescue the effects of DHODH knockdown in proliferation. N = 3. (C) qPCR of DHODH, 47S pre-rRNA and ACTIN in LN229 after 3 days of transfection with 2 different siRNA for DHODH. N = 2–6. (D) Western blot of DHODH in LN229 after 3 days of transfection with 2 different siRNA for DHODH or treated with 0.01 μM or 0.1 μM brequinar for 24 h. Fresh media with drugs were replaced the day after siRNA transfection or 24 h before harvesting for the brequinar-treated cells. (E) Amounts of brequinar, UMP, UDP, UTP and uridine in the LN229 measured by LC-MS/MS after 3 days of transfection with 2 different siRNA for DHODH or treated with 0.01 μM or 0.1 μM brequinar for 24 h. Fresh media with drugs were replaced the day after siRNA transfection or 24 h before harvesting for the brequinar-treated cells. Numerical values for each of the experiments represented are available in S8 Data. (PDF) [file pgen.1009117.s003.pdf]

**A**

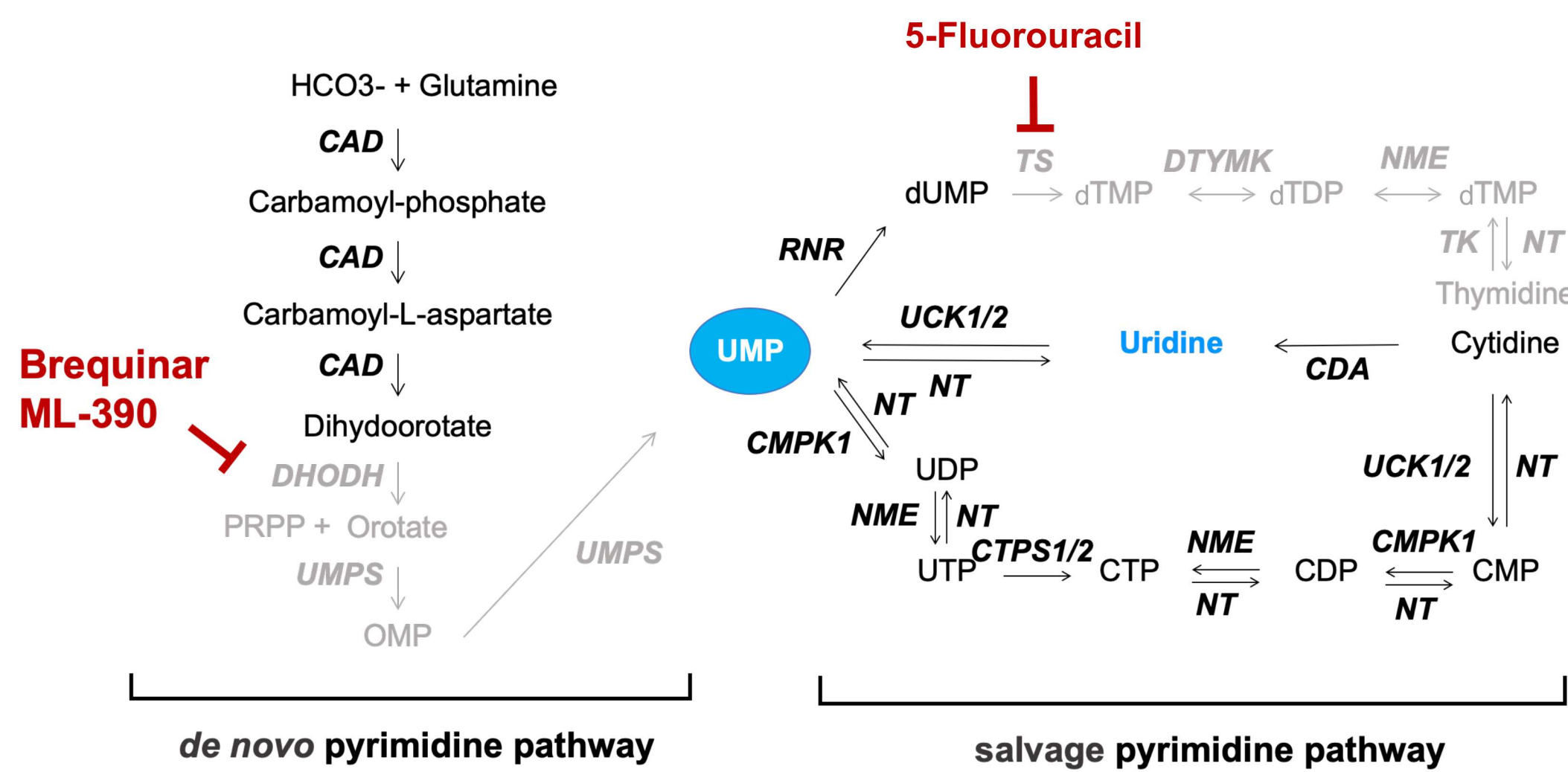

# B

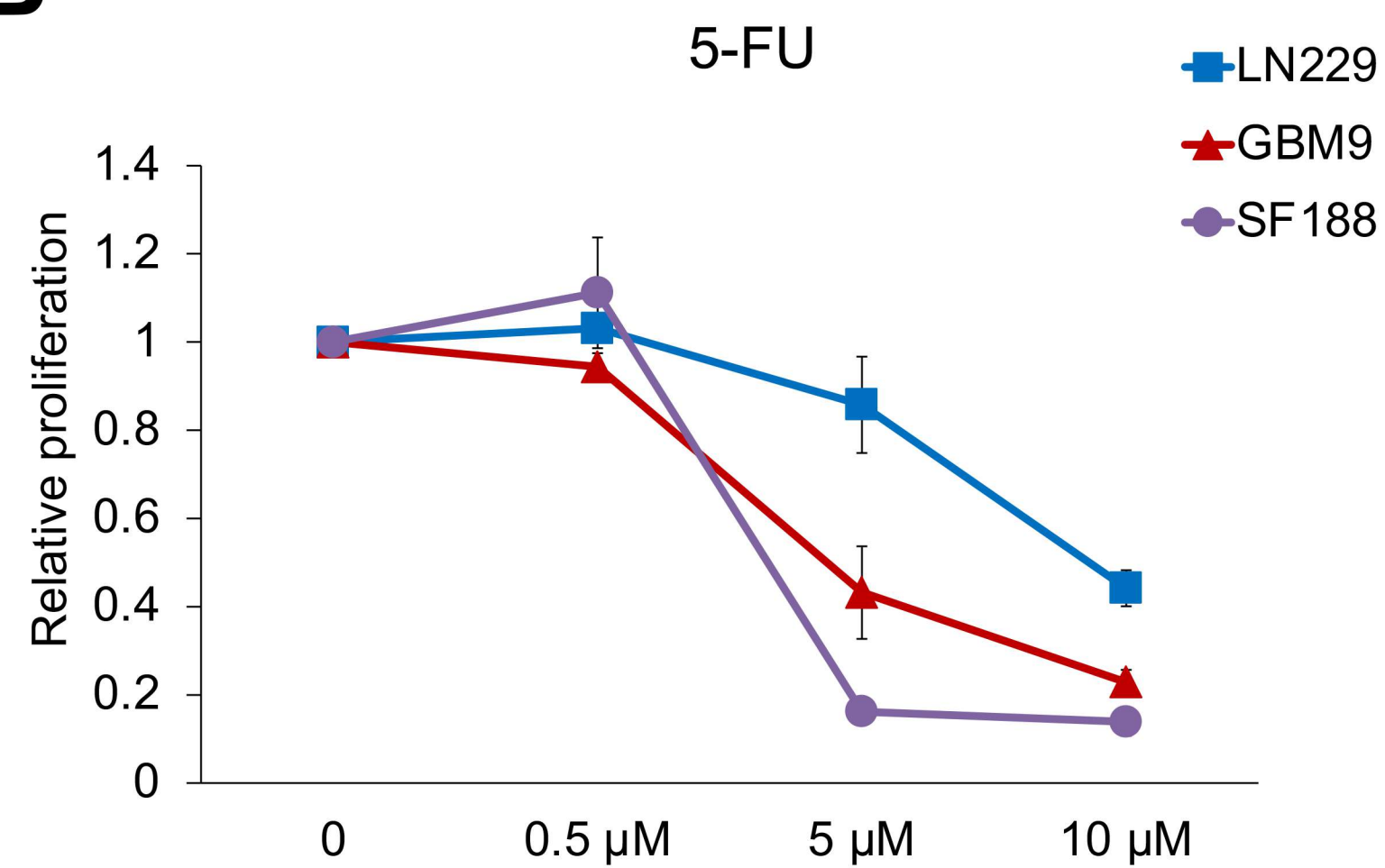

C

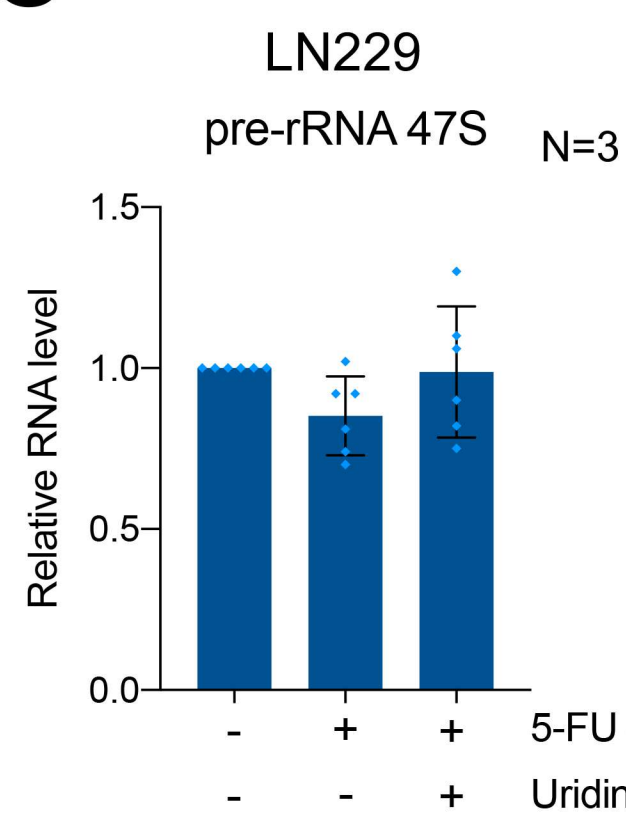

**D**

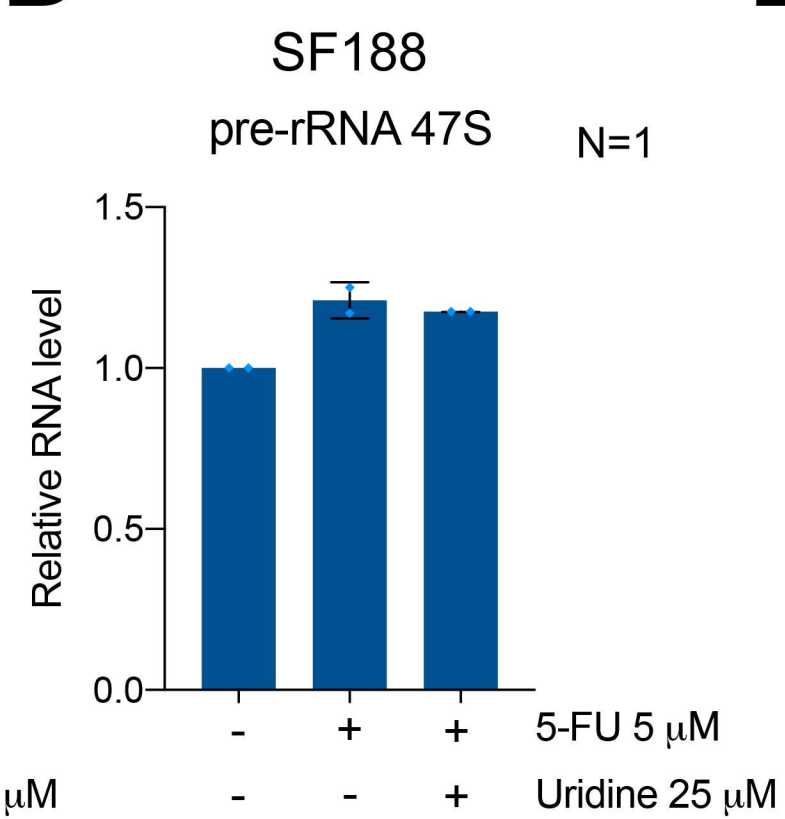

# E

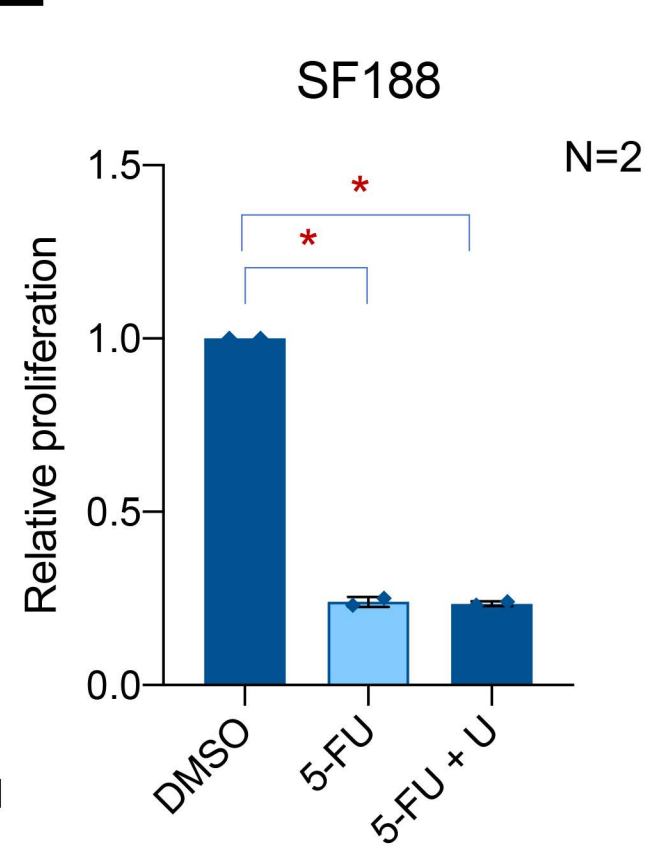

Figure S4

Supplement: S4 Fig — (A) Schematic representation of the action of the pyrimidine inhibitors 5-fluorouracil (5-FU, a TS inhibitor), brequinar, and ML390 (DHODH inhibitor). (B) Proliferation curve of increasing amount of 5-FU for the LN229, GBM9 and SF188 cells. Media with drugs were changed every 2 days for 6 days. (C, D) qPCR of 47S pre-rRNA normalized by ACTIN mRNA amount in LN229 (C) and SF188 (D) glioblastoma cells with or without 5-FU and with or without uridine for 24 h. 5-FU did not affect the production of pre-rRNA. Media with drugs were changed the day after seeding. (E) Relative proliferation of the SF188 glioblastoma cells with or without 5 μM 5-FU and with or without 25 μM uridine. Proliferation was assessed by crystal violet staining after 4 days of treatment. Uridine did not rescue the effects of 5-FU in proliferation. Numerical values for each of the experiments represented are available in S9 Data. (PDF) [file pgen.1009117.s004.pdf]

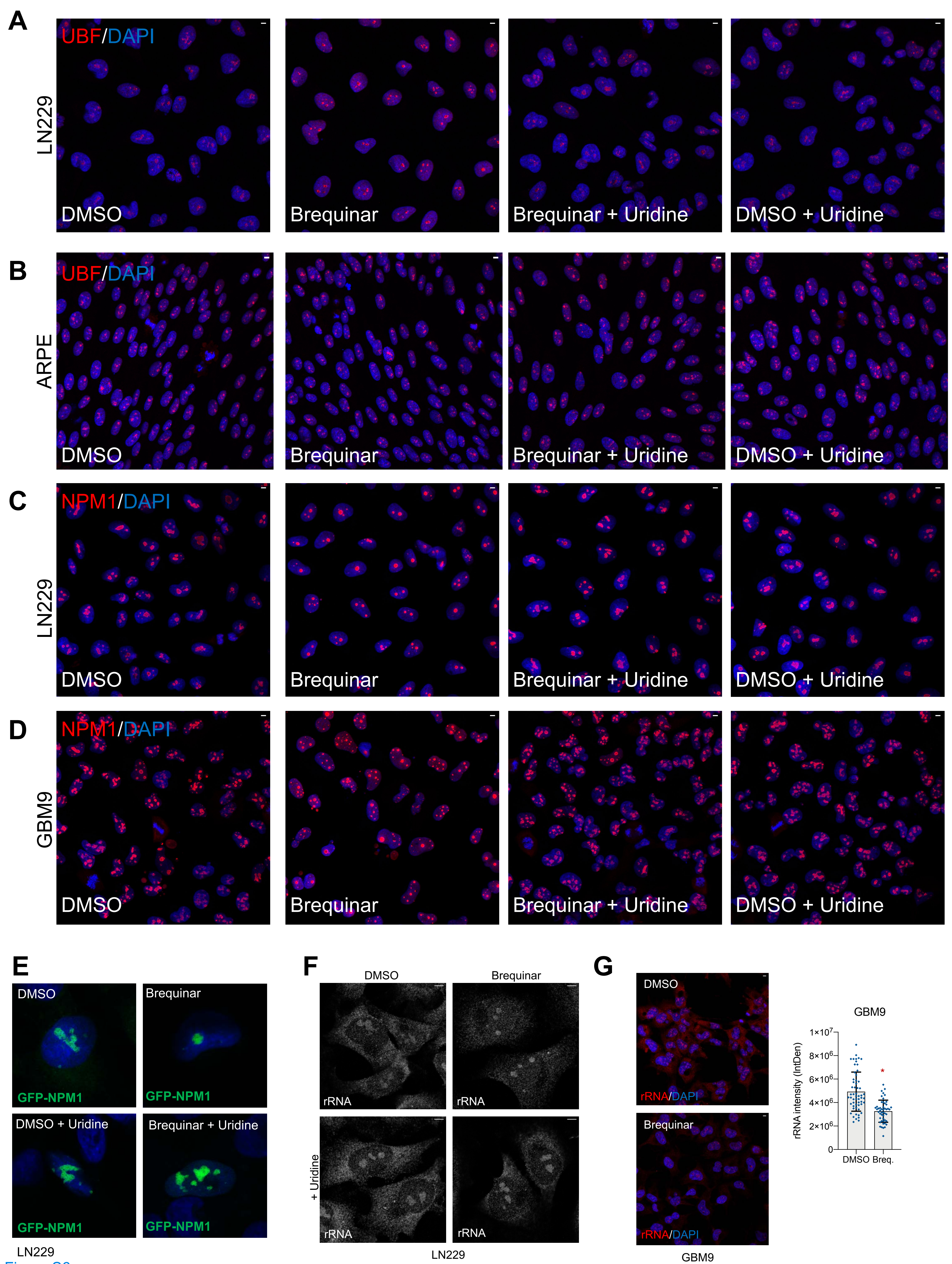

Supplement: S6 Fig — (A) Immunofluorescence of the rDNA transcriptional factor UBF in LN229 cells with or without 0.1 μM brequinar and with or without 100 μM uridine for 24 h. UBF clustered in the edges of the nucleolus (indication of nucleolar stress) upon brequinar treatment, which was rescued by the addition of uridine. Scale bar is 5 μm. (B) Immunofluorescence of UBF in ARPE with or with of 0.1 μM brequinar and with or without 100 μM uridine for 24 h. UBF did not cluster in the edges of the nucleolus upon brequinar treatment. (C) Immunofluorescence of the rRNA processor nucleophosmin 1 (NPM1) in LN229 cells with or without 0.1 μM brequinar and with or without 100 μM uridine for 24 h. NPM1 redistributed to the edges of the nucleolus (indication of nucleolar stress) upon brequinar treatment, which was rescued by the addition of uridine. (D) Immunofluorescence of NPM1 in GBM9 cells with or without 0.5 μM brequinar and with or without 25 μM uridine for 24 h. NPM1 redistributed to the edges of the nucleolus (indication of nucleolar stress) upon brequinar treatment, which was rescued by the addition of uridine. (E) Immunofluorescence of NPM1 GFP-tagged in LN229 cells with or without 0.1 μM brequinar and with or without 100 μM uridine for 24 h. NPM1 redistributed to the edges of the nucleolus (indication of nucleolar stress) upon brequinar treatment, which was rescued by the addition of uridine. (F) Immunofluorescence of rRNA in LN229 cells by using the anti-rRNA Y10b antibody with or without 0.1 μM brequinar and with or without 100 μM uridine for 24 h. Brequinar decreased the amounts of rRNA, which was rescued by uridine. (G) Immunofluorescence of rRNA in GBM9 by using the anti-rRNA Y10b antibody with or without 0.5 μM brequinar. Brequinar decreased the amounts of rRNA. Right panel shows the quantification by image J. For all the experiments, media with drugs and uridine were replaced the day after seeding. Numerical values for each of the experiments represented are available in S11 Data [file pgen.1009117.s006.pdf]
