# Supplementary material for: Inhibition of the de novo pyrimidine biosynthesis pathway limits ribosomal RNA transcription causing nucleolar stress in glioblastoma cells
Source: PLoS Genet. 2020 Nov 17;16(11):e1009117. doi: 10.1371/journal.pgen.1009117 (PMC7707548; doi:10.1371/journal.pgen.1009117)
Supplement: S5 Fig — (A) Representation of subcutaneous xenograft experiment with LN229 glioblastoma cells. Mice were treated with 15 mg/kg brequinar every 3 days by intraperitoneal injections (IP). (B) Representation of LN229 xenograft tumors from control and brequinar-treated mice with 10 mg/kg by daily IP injections. Tumor weight measurements of LN229 xenografts once the experiment was finished and the tumors harvested are indicated below each tumor picture. (C) Table showing the tumor and mouse weights of each mouse in the subcutaneous xenograft experiment. (D) qPCR of ACTIN mRNA levels normalized to same amounts of total RNA in the LN229 subcutaneous xenograft mice tumors. (E) Correlation of pre-rRNA, mature 28S and 18S rRNA, and ACTIN RNA levels with tumor weight. Only the pre-rRNA RNA levels in the control group showed significant correlation with tumor size. (F) qPCR of ACTIN mRNA levels normalized to same amounts of total RNA in the brain tissue of the mice used for the xenografts experiments in (B). Asterisks indicate p-values ≦0.05. Numerical values for each of the experiments represented are available in S10 Data. (PDF) [file pgen.1009117.s005.pdf]

A

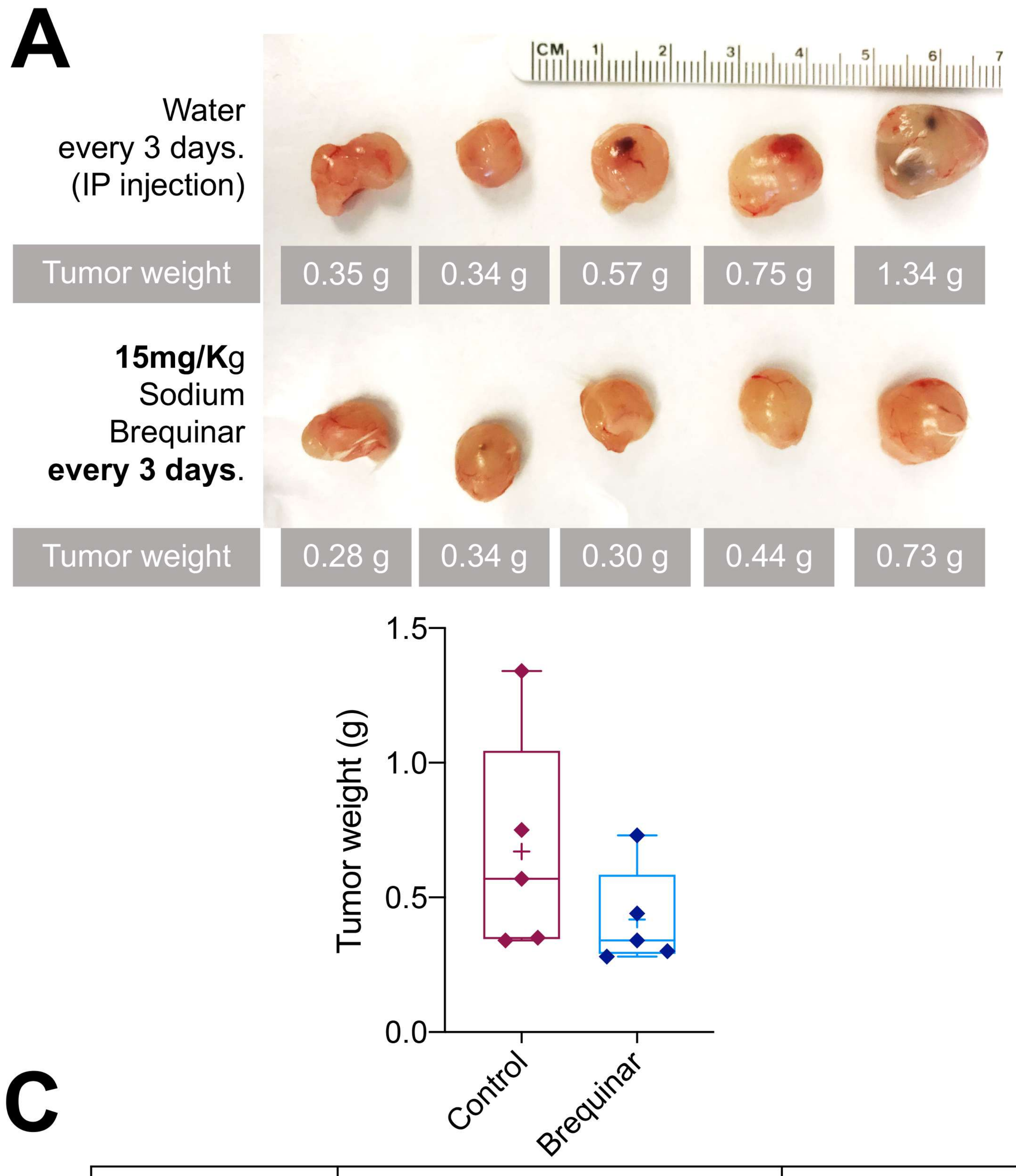

B

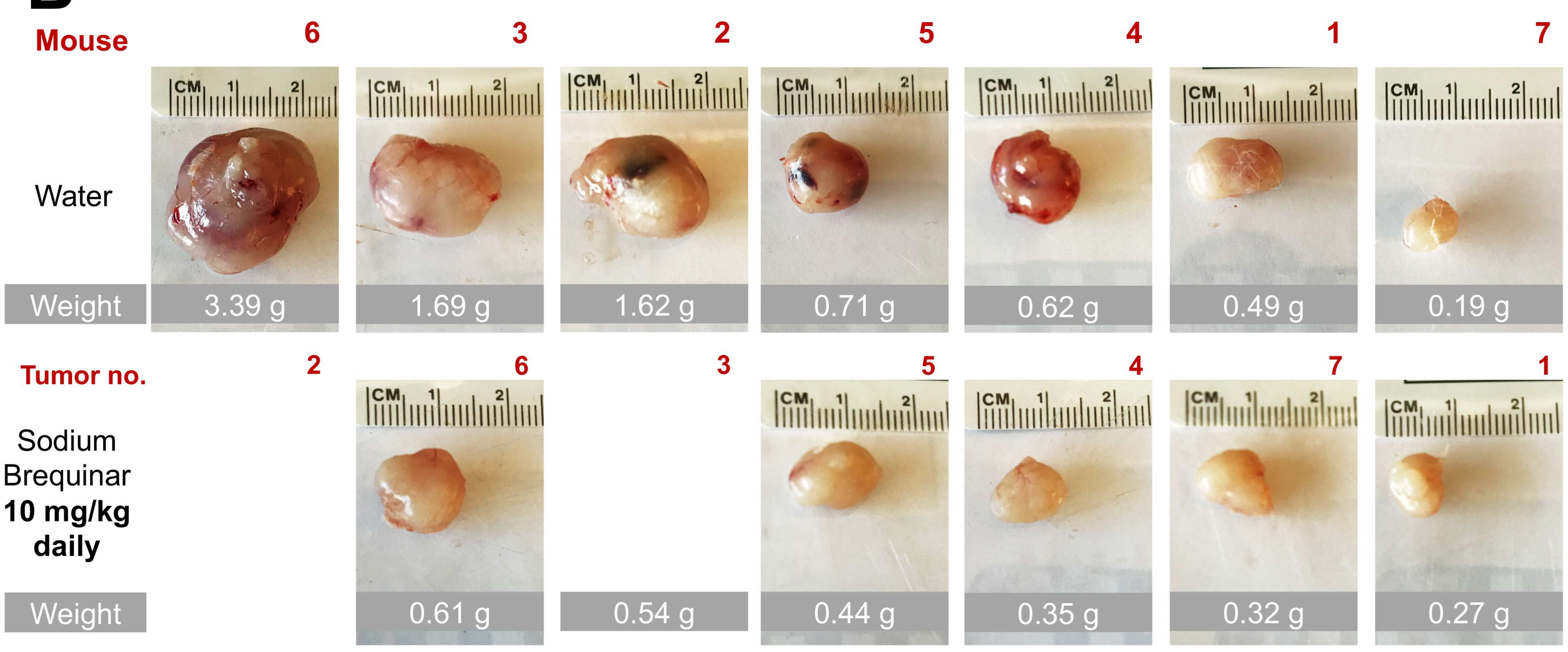

* Tumor #2 in brequinar group was an outlier.

** Picture of tumor #3 in brequinar group was not available.

C

Mouse	Tumor weight		Mouse weight		Δ (Mouse weight - Tumor weight)	
	Control	Brequinar	Control	Brequinar	Control	Brequinar
1	0.49	0.27	23.2	22.16	22.71	21.89
2	1.62	outlier	22.56	25.8	20.94	24.32
3	1.69	0.54	24.66	22.58	22.97	22.04
4	0.62	0.35	24.6	19.84	23.98	19.49
5	0.71	0.44	22.16	21.29	21.45	20.85
6	3.39	0.61	24.26	21.4	20.87	20.79
7	0.19	0.32	20.62	20.36	20.43	20.04
average	1.24	0.57	23.15	21.92	21.91	21.35
SD	1.10	0.42	1.49	1.96	1.32	1.60
p-value		0.16		0.21		0.49

D

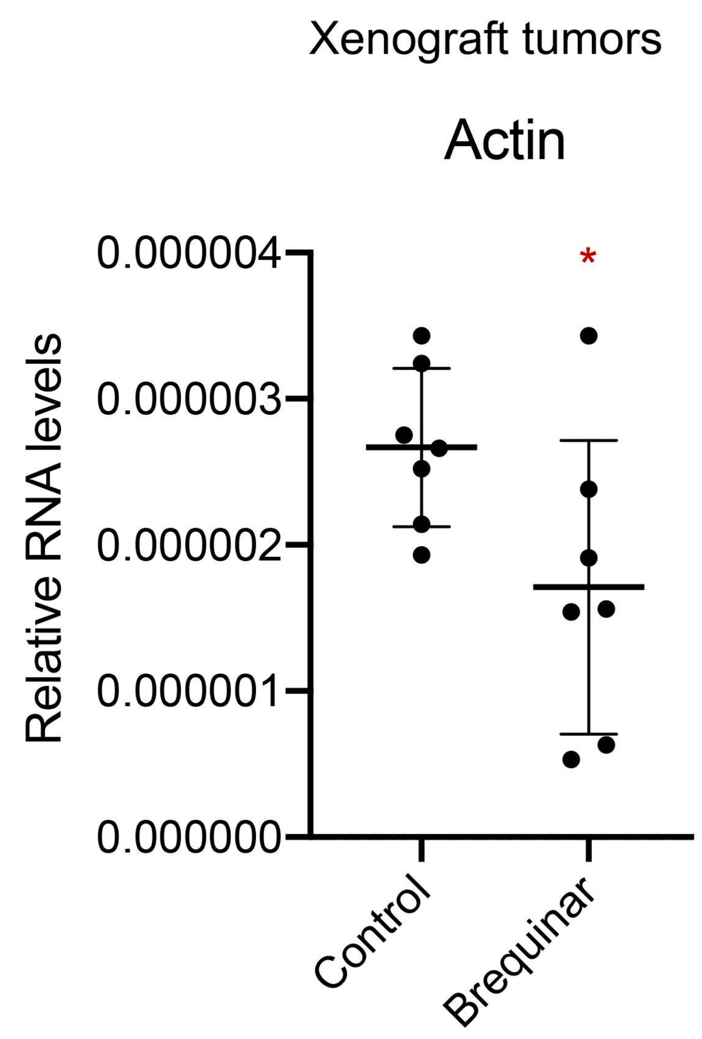

E

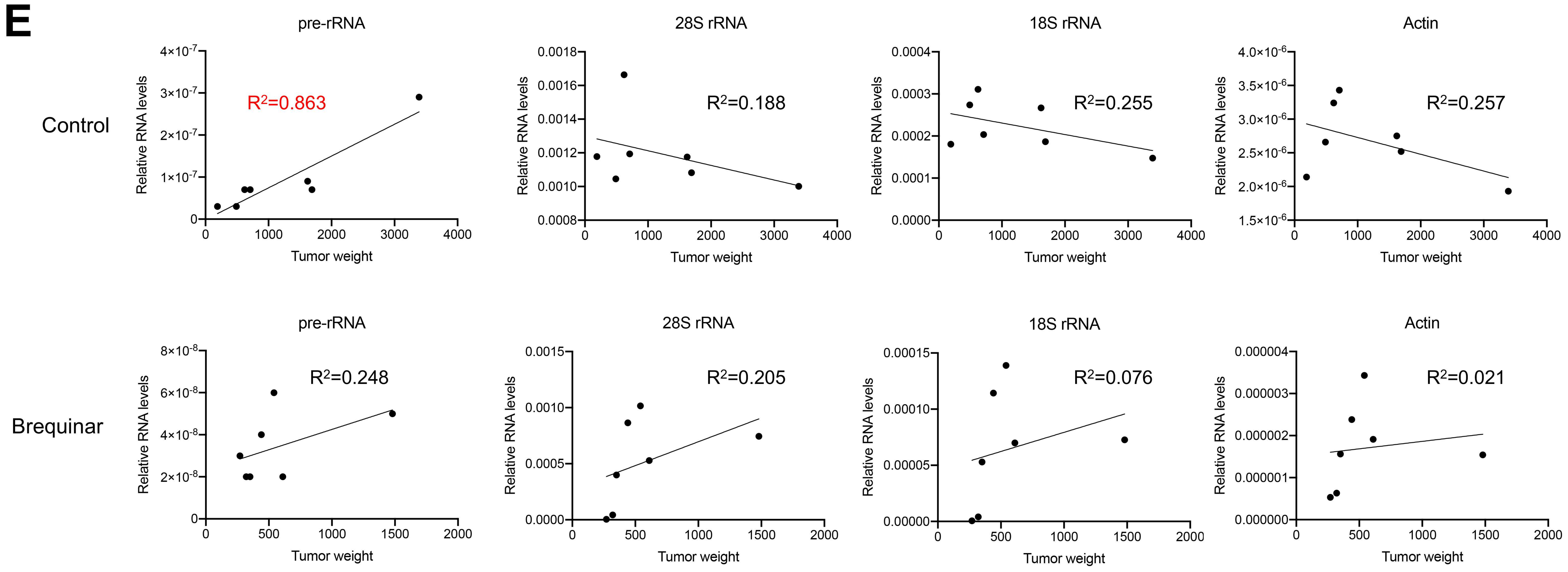

F

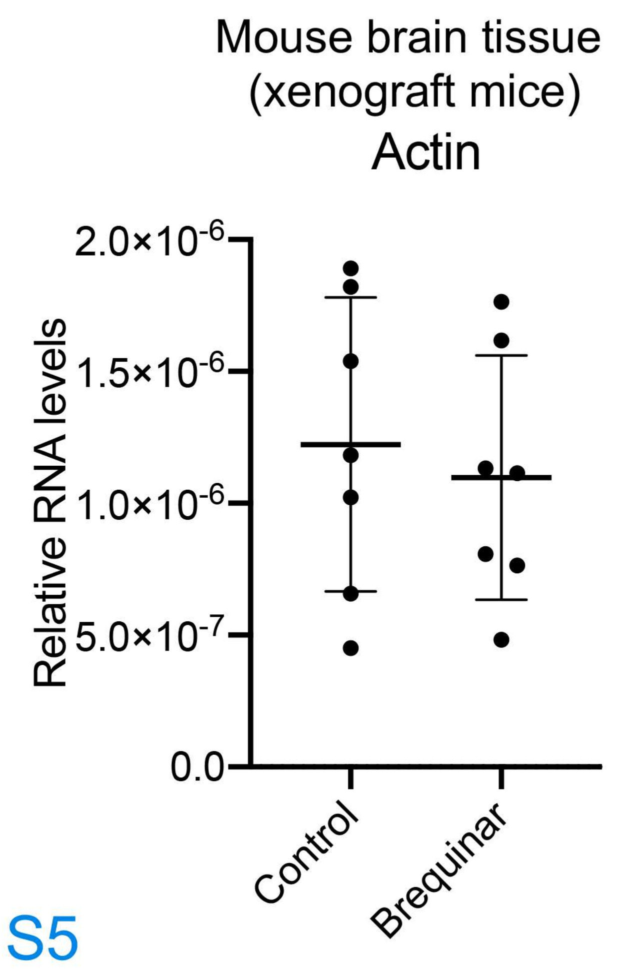

Figure S5
