# Supplementary material for: Inhibition of the de novo pyrimidine biosynthesis pathway limits ribosomal RNA transcription causing nucleolar stress in glioblastoma cells
Source: PLoS Genet. 2020 Nov 17;16(11):e1009117. doi: 10.1371/journal.pgen.1009117 (PMC7707548; doi:10.1371/journal.pgen.1009117)
Supplement: S1 Table — Table showing the amounts of brequinar, UMP, UDP, UTP and uridine in the LN229 tumor xenografts, brain tissue, liver tissue and serum in each mouse. (PDF) [file pgen.1009117.s007.pdf]

S1 Table. **Brequinar reduces the levels of pyrimidine nucleotides specifically in glioblastoma tumor xenografts tissues.** Table showing the amounts of brequinar, UMP, UDP, UTP and uridine in the LN229 tumor xenografts, brain tissue, liver tissue and serum in each mouse.

		UMP				UDP				UTP				Uridine				Brequinar			
		Xenog.	Plasma	Brain	Liver	Xenog.	Plasma	Brain	Liver	Xenog.	Plasma	Brain	Liver	Xenog.	Plasma	Brain	Liver	Xenog.	Plasma	Brain	Liver
Tumors		µg/g tissue	µg/ml plasma	µg/g tissue	µg/g tissue	µg/g tissue	µg/ml plasma	µg/g tissue	µg/g tissue	µg/g tissue	µg/ml plasma	µg/g tissue	µg/g tissue	µg/g tissue	µg/ml plasma	µg/g tissue	µg/g tissue	µg/g tissue	µg/ml plasma	µg/g tissue	µg/g tissue
Control	6	38.66	0.00	1.68	28.83	3.34	N/A	0.06	2.55	0.55	N/A	0.06	0.20	9.32	0.91	2.21	0.87	0.00	0.04	0.00	0.04
	3	48.73	0.36	2.29	24.70	8.58	N/A	0.10	2.77	0.45	N/A	0.03	0.15	6.05	1.41	1.72	0.74	0.00	0.13	0.00	0.01
	2	34.95	0.79	2.48	19.46	5.30	N/A	0.10	1.65	0.55	N/A	0.05	0.14	6.19	1.67	1.64	0.71	0.00	0.03	0.00	0.05
	5	48.52	N/A	2.20	24.48	9.23	N/A	0.08	2.88	0.72	N/A	0.04	0.15	7.24	N/A	1.78	0.92	0.00	0.05	0.00	0.09
	4	34.87	N/A	2.40	21.78	4.72	N/A	0.10	2.10	0.72	N/A	0.03	0.15	5.70	N/A	1.57	0.73	0.05	0.69	0.00	0.00
	1	46.88	N/A	5.30	14.96	9.80	N/A	0.19	1.39	0.57	N/A	0.17	0.10	4.46	N/A	2.13	1.21	0.29	4.29	0.06	-0.57
	7	50.35	N/A	2.13	25.11	2.61	N/A	0.07	2.99	0.08	N/A	0.02	0.14	8.75	N/A	2.29	0.97	0.00	0.00	0.00	0.06
Brequinar	2	0.59	0.17	2.00	22.95	0.06	N/A	0.09	2.42	0.10	N/A	0.02	0.13	3.45	1.59	1.36	0.40	9.92	26.15	1.60	89.19
	6	15.40	N/A	1.60	17.15	0.54	N/A	0.09	1.36	0.04	N/A	0.02	0.04	2.17	N/A	1.35	0.37	7.44	20.15	1.14	74.65
	3	18.52	0.00	2.16	25.50	1.39	N/A	0.09	3.08	0.08	N/A	0.02	0.15	1.47	1.02	1.74	0.43	8.28	29.40	2.17	89.42
	5	5.12	1.90	1.98	23.60	0.08	N/A	0.08	2.35	0.02	N/A	0.03	0.10	3.42	0.92	1.53	0.39	8.00	27.15	1.24	54.33
	4	17.63	1.84	1.96	16.52	0.92	N/A	0.09	1.80	0.06	N/A	0.03	0.12	1.72	1.30	1.61	0.49	5.80	26.70	1.41	49.79
	7	16.01	N/A	1.96	17.95	0.80	N/A	0.09	1.85	0.07	N/A	0.03	0.06	1.79	N/A	1.86	0.52	4.92	24.45	2.41	57.50
	1	3.89	0.00	1.75	16.70	0.09	N/A	0.08	1.66	0.06	N/A	0.03	0.14	3.68	1.21	1.45	0.52	6.72	23.40	2.20	40.68
