# Supplementary material for: Inhibition of the de novo pyrimidine biosynthesis pathway limits ribosomal RNA transcription causing nucleolar stress in glioblastoma cells
Source: PLoS Genet. 2020 Nov 17;16(11):e1009117. doi: 10.1371/journal.pgen.1009117 (PMC7707548; doi:10.1371/journal.pgen.1009117)
Supplement: S2 Table — (PDF) [file pgen.1009117.s008.pdf]

S2 Table. **Cell lines, antibodies, siRNA, primers, and reagents used in the present manuscript.**

**Cell lines**	**Source**	**Identifier**
p14/ARF -/- human Astrocytes	Marc Diamond lab	N/A
ARPE	Sandra Schmid lab	N/A
LN229	Ralph DeBerardinis lab	N/A
SF188	Ralph DeBerardinis lab	N/A
GBM9	Sandeep Burma	CRG del Alcazar et al. 2014
**Antibodies**	**Source**	**Identifier**
DHODH	Proteintech	14877-1-AP
UMPS	Sigma	HPA036178-100UL
ACTIN	Cell Signaling	13E5, #4970
TUBULIN	Sigma	T6199-100UL
p53	Calbiochem	JA1308
p21	Santa Cruz Biotechnology	C-19
phopho S139-H2AX (γ-H2AX)	Abcam	phospho S139, ab11174
MGMT	Sigma Millipore	MAB16200
GFAP	Abcam	whole antiserum, ab7260-50
ACETYL-TUBULIN	Sigma	T7451-100UL
UBF	Santa Cruz Biotechnology	F-9, sc-13125
NPM1	Santa Cruz Biotechnology	0412, sc-47725
rRNA	Novus biologicals	Y10b, NB100-662SS
HIF1alpha	Novus biologicals	NB100-105
**Chemicals, nucleotides**	**Source**	**Identifier**
Brequinar sodium	Tocris	6196/50
Brequinar	Cayman chemical	24445
ML390	Cayman chemical	21395
5-Fluorouracil	Sigma	F6627
Temozolomide	Sigma	T2577
Uridine	Alfa Aesar™	AAA1522706
**Oligonucleotides for qPCR**		
**Gene**	**F sequence**	**R sequence**
human pre-rRNA 47S	GTCCCCTCGTCTCTCCTCTC	CAAGTCGACAACCACTGGAG
human and mouse rRNA 18S	CTGCCCTATCAACTTTCGATGGTAG	CCGTTTCTCAGGCTCCCTCTC
human and mouse rRNA 28S	TGTCGGCTCTTCCTATCATTGT	ACCCAGCTCACGTTCCCTATTA
human VEGFA	TCTTCAAGCCATCCTGTGTG	TGCATTCACATTTGTTGTGC
human beta Actin	GGACTTCGAGCAAGAGATGG	AGCACTGTGTTGGCGTACAG
mouse pre-rRNA 47S	GCGTCGTTGCTCACTCTTAG	CACACATCCACAAGGACCAC
mouse beta Actin	TGTTACCAACTGGGACGACA	GGGGTGTTGAAGGTCTCAAA
**siRNA oligonucleotides**	**Source**	**Identifier**
universal controls #1	Sigma mission	SIC001
universal controls #2	Sigma mission	SIC002
siRNA DHODH1-1	Sigma mission	SASI_Hs01_00246561
siRNA DHODH1-2	Sigma mission	SASI_Hs01_00246562
