# Supplementary material for: Inhibition of the de novo pyrimidine biosynthesis pathway limits ribosomal RNA transcription causing nucleolar stress in glioblastoma cells
Source: PLoS Genet. 2020 Nov 17;16(11):e1009117. doi: 10.1371/journal.pgen.1009117 (PMC7707548; doi:10.1371/journal.pgen.1009117)
Supplement: Attachment — Submitted filename: Plos Genetics rebuttal letter 08-31-2020 MLN[1].pdf [file pgen.1009117.s020.pdf]

Response to reviewers comments

We thank the reviewers for their comments and very helpful suggestions. We have taken all the comments and suggestions into consideration while revising our manuscript. We have carried out several additional experiments and made appropriate changes to the manuscript as suggested by the reviewers. We are confident that, with the addition of these experiments and clarifications, we are submitting a much-improved manuscript. Below are point-by-point responses to the reviewers' comments

Reviewer #1

Reviewer #1: Lafita-Navarro et al. report that glioblastoma cells, sensitive and resistant to the chemotherapeutic agent temozolomide (TMZ), display strong sensitivity to the inhibition of de novo pyrimidine synthesis. The authors demonstrate that the inhibition of the mitochondrial pyrimidine enzyme, DHODH, impairs the synthesis of ribosomal RNA and leads to nucleolar stress. Furthermore, pyrimidine depletion relocalizes the distribution of nucleolar proteins specifically in transformed cells but not in normal cells. DHODH inhibition decreases pyrimidine levels and the growth of glioblastoma tumor cells and interestingly decreases DNA repair mechanisms enabling the prevention of resistance mechanisms induced by TMZ.

Fundamentally, this study is not conceptually novel because of the emerging literature in the cancer metabolism field, demonstrating the potential therapeutic strategy of targeting DHODH in several cancers. However, this study sheds light on the role of pyrimidines in glioblastoma and is interesting from a translational standpoint.

<u>Our response:</u> We thank the reviewer for their review on our work. While there have been recent studies demonstrating the potential therapeutic strategy targeting DHODH, our work dissects the molecular implications of inhibiting the *de novo* pyrimidine synthesis pathway in glioblastoma. We demonstrate that glioblastoma cells rely on this pathway to efficiently synthesize ribosomal RNA (rRNA). In contrast, non-cancer cells (and tissues) do not depend on *de novo* pyrimidine synthesis pathway to fulfill their pyrimidine demands for rRNA production. Therefore, we characterized a novel mechanism used by glioblastoma cells to maintain the elevated production of rRNA through the activation of the *de novo* pyrimidine synthesis pathway.

Major comments:

Reviewer #1: This study is well-executed and provides interesting translational information on the impact of targeting pyrimidine synthesis in GBM. The authors demonstrate that the immature 47S pre-rRNAs are controlled by pyrimidine availability. The specificity of this regulation to pyrimidine levels seems arbitrary as it is also predictable that purine depletion should have the same impact on newly synthesized rRNA.

<u>Our response:</u> The reviewer is correct in that depletion of purine nucleotides also decreases the synthesis of pre-rRNA. In fact, while we were working in this study, a paper was published showing that inhibition of purine guanosine monophosphate biosynthesis, which synthesizes guanosine monophosphate by *de novo* purine biosynthesis pathway, decreases the production of rRNA and glioblastoma cell growth [1]. The work is mentioned in the introduction: "*Recently, inhibition of the purine guanosine monophosphate biosynthesis was shown to decrease the production of rRNA and glioblastoma cell growth [1].*", as well as in the discussion section: "*Our results on rRNA and nucleolar*

stress are in agreement with previous studies showing that de novo purine biosynthesis is necessary for rRNA synthesis and proliferation in glioblastoma [1]".

While it is predictable that depletion of nucleotides would affect the synthesis of RNA and DNA, we demonstrate that glioblastoma cells have a preference for the *de novo* pyrimidine biosynthesis pathway to fulfill its nucleotide demands. In contrast, non-transformed cells such as ARPE or brain and liver tissues do not depend on the *de novo* pathways to synthesize pyrimidines. This dependency of glioblastoma cells on the *de novo* pathway could provide a underappreciate therapeutic window which underscores the importance of our study. This is now clarified in the discussion section *"It is likely that normal tissues do not depend on de novo biosynthesis of pyrimidines to maintain the intracellular pyrimidine pool, and that the production of pyrimidine by the salvage pathway is sufficient to fulfill their pyrimidine demands when the de novo pyrimidine biosynthesis is inhibited in normal tissues.".*

Reviewer #1. While the treatment with Brequinar led to an increase in p53 levels, TMZ does not seem to affect p53. The mechanisms by which Brequinar induces a decrease in cancer cell proliferation are probably dependent on the tumor-suppressive functions of p53. The authors should measure the levels of p21, the canonical target of p53, and measure the cytostatic effects of TMZ and Brequinar on the GBM cell cycle.

Our response: We thank the reviewer for this comment. We have revised our manuscript to include additional experiments to clarify the mechanisms by which DHODH inhibitors reduce viability of glioblastoma cells including western blots for p53 and p21 and cell cycle analyses.

Western blots for p53 and p21 in cells treated with brequinar or another DHODH inhibitor (ML390), indicate that while an increase in p53 levels upon DHODH inhibition is observed in all cell lines tested, only LN229, but not SF188 or GMB9, show a parallel induction of p21 (Fig 2M-O). This result agrees with a mutation in p53 in the DNA binding domain (G266E) in SF188. The p53 status in GMB9, a patient-derived cell line, is unknown but is likely to be mutated as we failed to observe p21 induction in this line similar to SF188. The known mutation status of p53 is now indicated in a table characterizing each cell line used in this study (S1B Fig) and in the results section.

As the reviewer suggested, we also performed cell cycle analysis in LN229 upon 24-h treatment with brequinar and ML390 (in the presence/absence of uridine) or upon TMZ (Fig 2L). Brequinar and ML390 induced cell cycle arrest in S phase, and this was rescued by exogenous uridine. Our results are in agreement with previous results [2-4]. In contrast, TMZ induced cell cycle arrest in S/G2. In the conditions used for the cell cycle analysis, no quantifiable subG0 was observed, suggesting that brequinar, ML390 and TMZ have cytostatic effects on this cell line.

Moreover, because SF188 and GMB9 did not show an induction of p21 upon brequinar or ML390 treatment, we measured apoptosis by Western blotting of cleaved caspase 3 (Fig 2 M-O). Our results indicate that at high concentration of brequinar and ML390 (1 and 4 μM respectively), apoptosis was activated in all glioblastoma cell lines but not in non-transformed ARPE cells. However, lower concentrations of the brequinar and ML390 (0.1 and 2 μM respectively), which were used for our growth experiments, caused activation of cleaved caspase 3 in SF188 and GBM9.

These results suggested that brequinar and ML390 have a combination of cytostatic and cytotoxic effects that overall affect the proliferation of glioblastoma cells. This is also clarified in the text: "*Our results indicated that treatment with 0.1 μM brequinar activated apoptosis in SF188 and to a lesser extent in GBM9, but not in ARPE or LN229 (Fig 2M). Similarly, treatment with 2 μM ML390 just activated apoptosis in GBM9 but not in ARPE or LN229 (Fig. 2N). However, higher concentrations of brequinar and ML390 (1 μM and 4 μM, respectively), activated apoptosis in LN229, SF188 and GBM9, but not in ARPE. Altogether, these results suggest that brequinar and ML390 cause a combination of cytostatic and cytotoxic effects, leading to impaired cell proliferation specifically in glioblastoma cells.*"

Reviewer #1. The absence of pyrimidine depletion in the brain tissue of the mice in response to Brequinar treatment is remarkable. However, it raises the question of whether Brequinar spares other nonproliferative tissues. The authors should present the effects of Brequinar on other differentiated tissues. Even though brain cells uptake detectable amounts of Brequinar, it is intriguing that there are no significant effects on the steady-state levels of pyrimidine nucleotide. This result suggests that most of the brain cells depend on the pyrimidine salvage pathway rather than the de novo pathway to maintain their nucleotide pool.

Our response: We agree with the reviewer that the vulnerability of glioblastoma cells, but not normal cell and tissues, to DHODH inhibition was indeed interesting and is one of the most important take home messages of or study. We thank the reviewer for suggesting additional controls which have increased the rigor and the confidence on this observation.

As the reviewer suggested, we now include pyrimidine quantification in liver tissues of the same mice used for xenografts and treated with brequinar from which we had also collected brain tissue. Our results, now shown in Fig. 4, indicate that liver tissue does show a decrease in the overall abundance of pyrimidines despite of its very high brequinar levels (second to serum). In the mouse liver tissues, only uridine was decreased. Fig. 4 now shows that brequinar specifically decreases the abundance of pyrimidines in tumor xenograft tissues and not in serum, brain, or liver tissues.

Our results suggest that normal tissues have the ability to use the salvage pathway to fulfill the nucleotide demands when the *de novo* pyrimidine biosynthesis pathway is inhibited. This is now clarified in the discussion sections *"It is likely that normal tissues do not depend on de novo biosynthesis of pyrimidines to maintain the intracellular pyrimidine pool, and that the production of pyrimidine by the salvage pathway is sufficient to fulfill their pyrimidine demands when the de novo pyrimidine biosynthesis is inhibited in normal tissues.".*

Reviewer #1. The combination of TMZ with Brequinar does not show any synergistic or additive effects. However, the model presented in Figure 5 suggests otherwise. If Brequinar decreases the synthesis of pyrimidines and the mechanisms of DNA repair (MGMT), and that TMZ activates MGMT to trigger DNA repair, then the combination should improve the antiproliferative effects.

Our response: In response to the reviewer's concern, we have modified the model (new Fig 6) to better represent the conclusions of our work. The mechanisms by which TMZ causes decreased proliferation involve DNA damage, but also the decrease in the levels of MGMT (when the cells are still sensitive to the drug) [5, 6]. This is now clarified and cited in the text ("*It has been shown that TMZ, in addition to causing direct DNA damage, limits repair by downregulating MGMT expression in some glioblastoma cell lines [5, 6].*") as well as in our model (Fig 6). Brequinar also affected the levels of MGMT. If both TMZ and brequinar act at least in part through the same mechanism (decreasing MGMT levels), it is likely that the effects of combining both drugs are not as additive or synergistic as one would expect if the drugs target different pathways. Nonetheless, TMZ and brequinar treatment's effects on proliferation in the TMZ-sensitive cells are slightly stronger than either of the drugs used alone in SF188 (Fig 2J). This point is also highlighted in the text "*The TMZ-sensitive cells decreased their proliferation with TMZ and brequinar alone and the combination of the agents decreased it further (Fig 2J and S2H).*"

Importantly, TMZ is very potent at causing death of glioblastoma cells, therefore attempting to observe synergy between TMZ and other drugs is a challenge. We have performed experiments both *in vivo* (not shown) and *in vitro* to determine whether significant synergy can be obtained from the combination of TMZ and DHODH inhibitors and the results indicate that these drugs are not significantly synergistic. Yet, the fact that TMZ-resistant cells retain sensitivity to DHODH inhibitors is exciting, given that efficient therapies to treat patients that have developed resistance to TMZ do not exist. Additional in-depth studies using more cell lines and PDX models would be necessary to define

the therapeutic potential of brequinar in treating patients whose tumors have developed resistance to TMZ.

Reviewer #1. The decrease in GBM LN229 tumor vascularization induced by Brequinar treatment is intriguing. The authors showed that VEGFA expression is reduced in response to Brequinar. Does pyrimidine depletion also decrease HIF1-a expression in tumors?

Our response: We now show the expression levels of HIF1α in the tumor tissues by Western blot (Fig 3G). Our results indicate that HIF1α was not highly expressed in the control or brequinar-treated tumors, and that brequinar does not cause significant changes in the levels or HIF1 a. It is possible that altered HIF1α activity or alternative mechanisms are responsible for the decrease mRNA levels of *VEGFA* upon brequinar treatment in the tumor xenografts. This is now indicated in the text "*HIF1α, a transcription factor known to induce the expression of VEGFA in hypoxia [7], was not detectable in the control or brequinar-treated tumors (Fig 3G). It is possible that VEGFA expression was regulated by other mechanisms in the LN229 xenograft tumors.*"

Reviewer #1 Minor comments:
Fig S2G: Thymidylate synthase converts dUMP to dTMP. The schematic should be amended, as "TMP" should be dTMP, dTDP etc. through the rest of the salvage pathway.

Our response: We thank the reviewer for noticing this inaccuracy, in the revised manuscript we have replaced TTP, TDP, TMP to dTTP, dTDP and dTMP in the figures and text.

Reviewer #2

Reviewer #2: This study demonstrates that DHODH inhibitor Brequinar has a selective cytostatic effect on glioblastoma cells that rely extensively on de-novo pyrimidine synthesis. The authors hypothesize that the decrease in cell proliferation and/or viability of GBM cells exposed to Brequinar was due, at least in part, to decrease in ribosomal RNA production and nucleolar stress.
Overall, in my opinion, this hypothesis is supported by the data. I have the following suggestions to improve the manuscript.

Our response: We thank the reviewer for their positive comments on our work. We have addressed their suggestions which have significantly improved our study.

Reviewer #2. It would be good to ensure that this phenotype was not an off-target effect of Brequinar, by reproducing these key findings with knocking down the endogenous DHODH in these cells. In the current version of the manuscript, the authors examined the effects of DHODH knockdown only on the cell number. They can also examine whether DHODH knockdown causes nucleolar stress similar to Brequinar by UBF and NPM1 labeling. In addition, the amount of rRNA in DHODH knockdown can be measured by qPCR and Y10b immunofluorescence. The effectiveness of DHODH siRNA knockdown (Fig. S2D) needs to be verified by western blotting.

Our response: We thank the reviewer for this comment as the new results have clarified that inhibition of DHODH activity is not equivalent to *DHODH* knockdown. As the reviewer suggested, we measured the abundance of pre-rRNA in cells where *DHODH* was silenced. *DHODH* knockdown by siRNA did not alter the abundance of pre-rRNA (S3C Fig) or the levels of pyrimidine (S3 Fig). These results suggested to us that partial knockdown does not mimic enzymatic inhibition of DHODH. This interpretation was supported by an experiment showing that treating cells with 0.01 μM of brequinar had no effect on pyrimidine levels (S3 Fig), thus suggesting that reduced levels of DHODH can be compensated by cancer cells. In a recent manuscript, we demonstrate that modest downregulation in DHODH expression, by knocking down its transcriptional activator AHR, does reduce the levels of

pyrimidines in glioblastoma cells, while it does in normal Rat fibroblast [11]. Thus, suggesting that glioblastoma cells are wired to maintain DHODH activity for their growth and a robust inhibition is required to limit DHODH activity.

To ensure that the effects of brequinar on pre-rRNA were specific, we performed qPCR measurements of pre-rRNA in cells treated with another DHODH inhibitor named ML390 in the presence or absence of uridine. The fact that uridine restores the levels of rRNA reduced by brequinar and ML390 indicates that both brequinar and ML390 effects on the abundance of pre-rRNA are due to depletion of pyrimidines. Importantly these inhibitors have been shown by others to specifically block DHODH activity [8, 9].

It is possible that cell death caused by knocking down *DHODH* is due to impairment in the mitochondrial electron transport chain, another function performed by DHODH. Our results are in agreement with previous work [10] showing that uridine does not rescue the decreased proliferation caused by *DHODH* knockdown in HeLa cells. This is now discussed and described in our manuscrip: "*Consistent with the effects of the DHODH inhibitors, knocking down DHODH expression by siRNA in LN229 and GBM9 cells decreased their proliferation. However, the addition of uridine to the media, did not rescue this effect (S3A and S3B Fig) as previously shown [10].*"

As requested by the reviewer, we have also added Western blots for DHODH in cells were DHODH was knocked down (S3A and S3D Fig).

Reviewer #2. Measurements of pre-processed rRNA (47S) and total (18S and 28S) by qPCR: the description of qPCR analysis is quite sparse and does not contain important details such as the number of replicates and the analysis method. For instance, why is the Y scale (relative RNA level) different in Fig. 1 E-F versus Fig. 3 G and J; how were these data normalized? In addition, I could not find primer sequences in the current version of the manuscript.

Our response: We thank the reviewer for this comment. We now have expanded the methods to clarify how the qPCR assays were performed ("***RT-qPCR*** *Total RNA was extracted with TRI Reagent® solution (Sigma) following the manufacturer's instructions, and cDNA was produced with the iScript RT Supermix for RT-qPCR (BIO-RAD). RNA levels were measured by quantitative PCR with the iTaq™ Universal SYBR® Green Supermix (BIO-RAD). Expression levels for each locus were normalized to β-actin expression. β-actin levels were normalized to RNA load for the cDNA production. The primers used in this study are indicated in Supplementary Table 1.*"). We have also included additional information in the figure legends to clarify how the RNA data was normalized. Figure 1 levels are related to the DMSO control after being normalized to actin levels. In the current Fig 4B and 4D, RNA levels are the values normalized to actin levels in each of the tumors.

Reviewer #2: Crystal violet staining quantifications were used in multiple experiments to infer relative proliferation (Fig. 2A, C,E,F,J,K; S2A,B,D,E,F,H,K,L). How was the crystal violet staining quantified (i.e. absorbance, manual colony counting, something else), and how was it normalized? Moreover, it is hard to interpret whether the effects of drug treatments were cytostatic or cytotoxic (i.e. causing a decrease in cell proliferation and/or cell death).

Our response: We thank the reviewer for this comment and have extended our entire methods section to contain more detailed information. We have also included additional information in the figure legends to clarify this.

"Cell proliferation *Six-well or twelve-well plates were seeded with 1.5 x 10^5 or 50,000 cells respectively. The day after seeding, the media was replaced with fresh media containing the appropriate inhibitors and metabolites. Media with drugs and metabolites was changed every 2 days for 6 days for the experiments shown in Figure 2A, and 2B and Figure S2A, S2B, and S2C. For the experiments shown in Figure 2D, 2E, 2I, and 2J and Figure S2D, and S2E, media with inhibitors and*

metabolites was replaced the day after seeding, and cell proliferation was measured 4 days later. Cell proliferation was measured by crystal violet staining and 595 nm absorbance quantification of the solubilized die. Values are represented as relative growth rate after normalizing by the control condition. Experiments were performed a minimum of 3 times."

In addition, we performed cell cycle analysis of LN229 cells treated with brequinar or ML390 in the presence/absence of uridine, or TMZ for 24 h. Brequinar and ML390 induced cell cycle arrest in S phase, and this was rescued by adding exogenous uridine. In contrast, TMZ induced cell cycle arrest at S/G2. In the conditions used for the cell cycle analysis, no quantifiable subG0 was observed suggesting that brequinar, ML390 and TMZ had cytostatic effects in this cell line. Our results in LN229 are in agreement with previous results [2-4].

We also measured apoptosis for cleaved caspase 3 by Western blotting (Fig 2 M-O). Our results indicated that at high concentration of brequinar and ML390, apoptosis was activated in all glioblastoma cell lines but not in non-transformed ARPE cells. However, low concentrations of the drugs just caused activation of cleaved caspase 3 in SF188 and GBM9.

These results suggested that brequinar and ML390 cause a combination of cytostatic and cytotoxic effects that overall affects the proliferation of glioblastoma cells, likely dependent on the landscape of oncogenes and tumor suppressors present in each cell. This is also clarified in the text ("*Altogether, these results suggest that brequinar and ML390 cause a combination of cytostatic and cytotoxic effects leading to impaired cell proliferation specifically in glioblastoma cells.*")

Reviewer #2: Y10b and p53 immunofluorescence labeling (Fig. 4 C-D) also needs to be quantified.

<u>Our response:</u> As suggested by the reviewer, we have included quantifications of rRNA and p53 immunofluorescences (Fig 5D and 5F, and S7G Fig).

Reviewer #2: Western blots in Fig. 2G-H and Fig. 2L can also use quantification of gamma-H2AX and p53 levels.

<u>Our response:</u> We have included quantifications of Western blot for gamma-H2AX, p53 and MGMT levels (S2F, S2G and S2I-J Fig).

Reviewer #3

Reviewer #3: Recent high-impact studies have drawn attention to the vulnerability of glioblastoma to inhibition of de novo pyrimidine synthesis (Refs. 10, 11). Consistent with that, the authors report that glioblastoma cells have active de novo pyrimidine biosynthesis. They show brequinar inhibition of DHODH in that pathway in GBM results in inhibition of ribosomal RNA synthesis and induces nucleolar stress. This treatment reduces glioblastoma proliferation, including in cells made resistant to the chemo agent temozolomide in vitro. The authors thus propose pyrimidine biosynthesis inhibition may resolve chemo resistance in GBM.

The writing is uniformly excellent and concise. The observation of reduced vascularization in xenograft tumors with brequinar seems interesting.

<u>Our response:</u> We thank the reviewer for their positive comment.

Reviewer #3: My overarching concern is that the study is built on previous findings implicating de novo pyrimidine biosynthesis being an Achilles heel in GBM rather than, say, performing genomic

experiments (or data mining) to nominate pathways or prioritize that for pyrimidine. There is weak or no justification for all the models used as stand-ins for human GBM, and different cells are used for different experiments with different controls without justification. Whereas all of us in this field struggle with the balance between biological relevance vs. effect size/penetrance (feasibility), this work seems to ignore that without accounting for it. With the possible exception of the mRNA expression data mining, there is little evidence this work is defining general aspects of GBM biology in cell culture, xenografts or natural cancer.

Our response:

Rationale for our study: The reviewer is correct that, while we were completing the experiments in this manuscript, other labs demonstrated that inhibiting DHODH causes glioblastoma cell death. We have cited these manuscripts in our study. Our interest in *de novo* pyrimidine biosynthesis pathway developed from a recent observation published by our lab in JBC. We discovered that the transcription factor aryl hydrocarbon receptor (AHR), which is necessary for glioblastoma growth, regulates the expression of the *de novo* pyrimidine synthesis enzymes DHODH and UMPS in glioblastoma cells [11]. In the current version of the manuscript, we describe our recent publication in the introduction section as the rationale for our interest in the importance of the *de novo* pyrimidine biosynthesis pathway in glioblastoma cells: "*Moreover, our lab recently discovered that Aryl hydrocarbon receptor (AHR) which is necessary for glioblastoma growth [9-11], mediates the expression of DHODH and UMPS in MYC-overexpressing fibroblasts and in glioblastoma cells [12].*".

Justification for the models used in our study: We randomly chose 3 glioblastoma cell lines from diverse origins to study the cell biological effects of DHODH inhibition. Our interest was to understand the general mechanism by which glioblastomas, independently of their genetic background, rely on the *de novo* pyrimidine synthesis pathway for growth. Therefore, we chose a diverse set of cell lines (pediatric/adult, female/male, active/inactive p53) as models to understand the molecular implications of targeting DHODH in a general setting. Given that all 3 cell lines caused very similar effects on growth, rRNA synthesis, and nucleolar stress, we believe that the effects of DHODH inhibitors are global and unaffected by the specific genetic background or each glioblastoma cell.

Our work supports recent studies from other labs on the dependence of glioblastoma to the *de novo* pyrimidine synthesis pathway [12, 13]. Furthermore, we describe the molecular implications of inhibiting DHODH activity in this cell type: reduced pre-rRNA synthesis and induction of nucleolar stress, which leads to reduce proliferation and growth of glioblastoma cells and tumors.

Reviewer #3: The selectivity of the primary agent is suggested to be supported by a second inhibitor of the same target, but the evidence is not convincing. They created a drug resistance model, but it is not clear to me if what they achieved is valid in the context of human chemo resistance.

Our response: We have extended the experiments performed with the DHODH inhibitor ML390. The revised manuscript now includes qPCR for 47S pre-rRNA and Actin, cell cycle analysis, and Western blots using both brequinar and ML390 (Fig 1G and 1H, and Fig 2L-O). By performing these experiments, we now demonstrate that effects of inhibiting DHODH by brequinar and ML390 are essentially the same.

Regarding the temozolomide-resistant cells, one of the best-known explanations of acquired resistance to temozolomide in glioblastoma patients is the increase expression of MGMT, which results in reduced DNA damage [14]. We show that the *in vitro* generated temozolomide resistant cell line dramatically increases the levels of MGMT and consistently, decreases DNA damage as shown by decrease gamma-H2AX (Fig 2K). Therefore, while the cell line was generated *in vitro*, the molecular characteristic of temozolomide-resistant glioblastoma cells recapitulates the molecular characteristics of temozolomide-resistant glioblastoma tumors.

Reviewer #3: The back and forth between proliferation, apoptosis, p53, DNA repair, etc. was confusing. I have no problem thinking about their complex interconnections, but there should first be some context and explanation of the relevance of each and how they are being interpreted to interact. Here these aspects seemed to be used almost interchangeably to make one major argument.

Our response: We have revised the manuscript to more systematically describe the effects of DHODH inhibitors on rRNA, reduction in cell growth and nucleolar stress (measured by UBF and NPM1 redistribution and p53 upregulation). Moreover, we have now included cell cycle analysis in cells treated with brequinar, ML390 and TMZ and we performed Western blots showing cleaved caspase 3 as a readout of activation of apoptosis in cells treated with brequinar or ML390 in all tested lines.

Our recent results led to the conclusion that the inhibition of DHODH results in a combination of cytostatic and cytotoxic effects that overall decrease the proliferation and growth of glioblastoma cells and tumors. We have clarified this in the text: "*Altogether, these results suggest that brequinar and ML390 cause a combination of cytostatic and cytotoxic effects leading to impaired cell proliferation specifically in glioblastoma cells.*"

Major concerns

Reviewer #3: It is not clear how the analysis of mRNA expression was done (e.g., what normalization approach, which algorithm).

Our response: We thank the reviewer for this comment. We now have expanded the methods section to clarify how the mRNA assays were performed: "***TCGA and CGGA data analysis:*** *RNA expression data for CAD, DHODH, and UMPS in grade II to IV (GBM) gliomas was obtained from the RNA-seq experiments deposited in The Cancer Genome Atlas (TCGA) Program through the Pancancer TCGA project (https://portal.gdc.cancer.gov) and the Chinese Glioma Genome Atlas (http://www.cgga.org.cn). TCGA-GBM and TCGA-LGG FPKM gene expression and clinical data were downloaded using the R package 'TCGABiolinks' [15].*

RT-qPCR: *Total RNA was extracted with TRI Reagent® solution (Sigma) following the manufacturer's instructions, and cDNA was produced with the iScript RT Supermix for RT-qPCR (BIO-RAD). RNA levels were measured by quantitative PCR with the iTaq™ Universal SYBR® Green Supermix (BIO-RAD). For the experiments in vitro, expression levels for 47S pre-rRNA, 18S rRNA and 28S rRNA were normalized to β-actin expression. β-actin levels were normalized to RNA load for the cDNA production. Relative RNA levels are shown as a ratio between the experimental and control conditions. For the in vivo experiments, expression levels for 47S pre-rRNA, 18S rRNA, 28S rRNA and VEGFA are shown for each tumor after normalizing the expression levels of each locus to β-actin mRNA levels. β-actin levels were normalized to RNA load for the cDNA production. The primers used in this study are indicated in Supplementary S2 Table.*"

Reviewer #3: Methods and statistics were very, very minimally described. For example, I couldn't find descriptions or sources of any of the antibodies.

Our response: We thank the reviewer for this comment. We now have expanded the methods section to better describe the techniques, source of the reagents, and data processing used in this work. All antibodies, primers and reagents used in the study are presented in Supplementary S2 Table**.**

Reviewer #3: Three cell lines are used as cell culture GBM models. Why these cell lines? On what basis was one of those chosen for the xenograft studies? What is known genetically about these, particularly oncogenic pathways? In addition to refs., a suppl. table of that would be useful.

<u>Our response:</u> See our response above and <u>Supplementary S2 Table</u> for a description of all cell lines used in this study. We have included a table showing the origin as well as genetic information of each of the cell lines used in the manuscript (S1B Fig). LN229 was chosen for xenografts because according to our proliferation assay, they were most sensitive to low amounts of brequinar *in* vitro (Fig 2A).

Reviewer #3: What was rationale for that control cell (normal human p14ARF-/- immortalized) vs., say, stage I, II and III glioma?

<u>Our response:</u> We have clarified this concern. We now include a table with information on all the cell lines used in this study (S1B Fig). In addition, we explain in the text that astrocytes are differentiated non-transformed glial cells and therefore present a suitable model for our *in vitro* studies *"…to normal human p14ARF-/- immortalized astrocytes which are non-transformed differentiated glial cells (Fig 1B)"* which we used as controls.

Reviewer #3: Re:previous two items: whether it was convenience or absolute necessity that resulted in chosen cell lines and controls, the authors should provide rationale for their study design, up front in Intro or Results.

<u>Our response:</u> We thank the reviewer for this suggestion. We have now established a rationale for our study design and use of chosen cell lines. We consistently included additional information on the cell lines used throughout the text as well as in the figures.

<u>Introduction section now says:</u> **"***Moreover, our lab recently discovered that Aryl hydrocarbon receptor (AHR) which is necessary for glioblastoma growth [9-11], mediates the expression of DHODH and UMPS in MYC-overexpressing fibroblasts, and in glioblastoma cells [12]"*

<u>Results section now says:</u> "*To investigate the need of glioblastoma cells for the de novo pyrimidine biosynthesis pathway, we chose 3 distinct cells line (S1B Fig): SF188, a commercially available pediatric male cell line with mutated p53 in the DNA binding domain (G622E); LN229, a commercially available adult female cell line with mutated p53 outside the DNA binding domain (P98L); and the patient derived GBM9 with unknown p53 mutation status [16].*"

Reviewer #3: There is no description of western blotting methods, including signal detection. The signal is much lower for the control actin mRNA in the control cell (normal human p14ARF-/- immortalized) and saturated for the test cells.

<u>Our response:</u> We now have expanded the methods section to better explain the techniques and processing used in this study. In addition, we have provided quantification of the Western signals for Fig 1B.

Reviewer #3: It was claimed that they "confirmed that DHODH and UMPS protein levels were higher in GBM cell lines (LN229, GBM9 and SF188) compared to normal human p14ARF-/- immortalized". The latter showed no expression of the test mRNAs. This suggests the proper experiment would be different stages of glioma. I don't object to doing such experiments, but the information has little usefulness without more context.

<u>Our response:</u> Our results indicate that glioblastomas have increased levels of DHODH and UMPS in comparison with normal non-transformed glial cells (astrocytes). We have corrected the text to accurately reflect our data shows: "Western blot of LN229, GBM9, SF188 and human p14ARF-/-

immortalized astrocytes (S1B Fig.), which are non-transformed differentiated glial cells, showed that DHODH and UMPS protein levels were higher in glioblastoma cells (Fig 1B) [27]"

Reviewer #3: What was rationale for using retinal epithelial ARPE cells as controls?

Our response: We have chosen to use ARPE as an additional control cell line because this is a spontaneously immortalized cell line and used by our lab [17-19], and other labs [20, 21], serving as a good normal cell model. Using ARPE and astrocytes as controls, as well as normal brain and liver tissues, we confirmed that glioblastoma cancer cells have higher sensitivity than normal non-transformed cells, which has been also shown by others [13].

Reviewer #3: Cell culture concentrations of brequinar and ML-390 were determined empirically here, but this is a critical issue that would be important to compare to other cell line publications, especially the latter which only shows an effect in uM concentration.

Our response: We thank the reviewer for calling our attention to this issue. We have now clarified in the text that the concentrations of brequinar and ML390 used in this study are based on previous publications, which are cited in the text. " *Concentration ranges of brequinar and ML390 used to treat glioblastoma cells were selected based on previous studies in leukemia cells [8].*"

Reviewer #3: Suppl. data do not show experiments and results clearly stated in Results. E.g. 1, "First, we measured the proliferation of non-transformed human p14ARF-/- astrocytes, and retinal epithelial ARPE cells as controls, and the GBM cells LN229, GBM9, and SF188 over 6 days in the presence of increasing amounts of brequinar (Fig 2A)". But S2A did not have GBM9 data.

Our response: We have made it clearer in the text which figures refer to which cell lines. "*First, we measured the proliferation of non-transformed cells (human p14ARF-/- astrocytes (Fig 2A) or retinal epithelial ARPE cells (S2A Fig))".*

Reviewer #3: [#8 Cont'd]. E.g. 2: "GBM cells were more sensitive to brequinar compared to ML-390, based on activation of apoptosis measured by cleaved caspase 3 and PARP1 (S2C Fig)." But Fig S2C does not show data for GBM9 and does not compare LN229 and SF188 to any other cell. More problematic, the untreated cell cleaved PARP1 and Casp3 look very similar to 2uM ML-390 treated LN229 and SF188. The sensitivity of GBM to brequinar and ML-390 is said to be higher but that is not shown for GBM9/ML-390 and the effect of GBM9/brequinar is intermediate to that for the other GBM cells and astrocytes. That suggests there is not a general GBM-pyrimidine pathway association, and, if there is, GBM9 is anomalous. And it raises questions about the in vitro efficacy of ML-390 (this is especially surprising to leave an open question when the investigators measured levels of UMP, UDP, UTP and uridine in the cells by LC-MS/MS for brequinar).

Our response: We thank the reviewer for pointing out this issue. We have now replaced the previous cleaved capase 3 experiment for a much expanded Western blot section. We compared the activation of apoptosis by looking at cleaved caspase 3 in ARPE, LN229, SF188 and GBM9 treated with brequinar or ML390 (Fig 2 M-O). Our results indicate that both brequinar and ML390 activated apoptosis as observed in the Western blot for cleaved caspase 3 at high concentrations (1 and 4 μM, respectively). However, lower concentrations of brequinar and ML390 (0.1 and 2 μM, respectively) only caused apoptosis in SF188 and GMB9 but not in ARPE or LN229. It is likely that the different genetic backgrounds accounts for this variability.

In addition, as the reviewer suggested, we have included LC-MS/MS measurements of pyrimidine levels in GMB9 cells treated with the other DHODH inhibitor ML390 (Fig 1F). Our results indicated that ML390 also decreased the levels of pyrimidines in GMB9 cells.

Reviewer #3: There are several problems. It is suggested brequinar and ML-390 somehow cross-validate specificity of pharmacological effects, but they don't do so convincingly. It is suggested proliferation is inhibited but mechanistic evidence is apoptosis; and is not convincing (preceding comment).

Our response: In the revised manuscript we included several experiments cross validating the the two DHODH inhibitors in rRNA expression and cell growth in all 3 cell lines. We have now icell cycle analysis of glioblastoma cells treated with brequinar or ML390 in the presence/absence of uridine. Brequinar and ML390 induced cell cycle arrest in S phase, and this was rescued in the presence of additional uridine. In the conditions used for the cell cycle analysis, no subG0 was observed, suggesting that brequinar and ML390 had cytostatic effects in this cell line. Our results in LN229 are in agreement with previous results [2-4].

We also measured apoptosis by Western blotting for cleaved caspase 3 (Fig 2 M-O). Our results indicated that at high concentration of brequinar and ML390 (1 and 4 μM, respectively), apoptosis was activated in all glioblastoma cell lines but not in non-transformed ARPE cells. However, low concentrations of the drugs (0.1 and 2 μM for brequinar and ML390, respectively) just caused activation of cleaved caspase 3 in SF188 and GBM9.

These results suggested that brequinar and ML390 cause a combination of cytostatic and cytotoxic effects that overall affects the proliferation of glioblastoma cells. This is also explained in the text "*Altogether, these results suggest that brequinar and ML390 cause a combination of cytostatic and cytotoxic effects leading to impaired cell proliferation specifically in glioblastoma cells.*"

Reviewer #3: It was stated that p53 levels did not increase with TMZ but did with brequinar. However, Fig 2G shows p53 increased with TMZ in LN229, but the figure lacks statistical analysis. That is also problematic because I could not find anywhere those 2G/H experiments were performed, say, in triplicate or were replicated in multiple experiments.

Our response: We have included quantifications of Western blot signals for γH2AX, p53 levels and MGMT levels (S2F, S2G and S2I-J Fig). In addition, more information on the methods and figures legends have been added to clarify the concerns raised by the reviewer. *"(F) Western blot quantification by Image J of Fig 2F and additional experimental replicates. (G) Western blot quantification by Image J of Fig 2G and additional experimental replicates. N=2….(I) Western blot quantification by Image J of γH2AX in Fig 2K and additional experimental replicates. The results are represented for each experimental replicate. N=2. (J) Western blot quantification by Image J of p53 and MGMT in Fig 2K and additional experimental replicates. N=2 for p53, N=3 for MGMT."*

Reviewer #3: The 2G/H experiments again show there is not a general GBM response to TMZ or brequinar according to the p53 effects in LN229 vs GBM9. I do not mean to suggest there should be a general GBM response. However, the authors repeatedly suggest that. It is problematic that a total of 3 cell lines are put forward to show this is not a single cell line study, but different experiments only show data for one or different combinations of two cell lines. They are compared to different controls for different experiments.

Our response: We have included quantifications of Western blot signals for p53 levels (S2F, S2G and S2I-J Fig) in all glioblastoma cell lines. In addition, we included additional Western blots comparing ARPE, LN229, SF188, and GMB9 treated with brequinar or ML390. Our results indicate that p53 levels consistently increase in LN229, SF188, and GMB9 when the cells are treated with the DHODH inhibitors brequinar or ML390.

Reviewer #3: The idea of generating in vitro chemo resistant cells is interesting. However, it is not clear to me what was done and why. Fig. 2J allows comparison of unselected vs. resistant SF188. It shows untreated resistant SF188 has 50% of proliferation rate vs. unselected. However, in Fig. 2K, all experiments for unselected and resistant show the untreated had a relative proliferation of 1.

Our response: Glioblastoma tumors frequently develop resistance to therapy with temozolomide due to increased expression of DNA repair enzymes that in turn leads to decreased DNA damage. We generated temozolomide resistant cells to study whether these were still sensitive to the pharmacological inhibition of DHODH with brequinar as a potential therapeutic alternative for glioblastoma. Current Fig 2H shows the process of generation of SF188 TMZ-resistant cells. Current Fig 2J shows relative proliferation normalized by the DMSO control in each group. We have included a similar graph normalizing all the data to the SF188 TMZ-sensitive DMSO control condition (S2H Fig) to clarify the reviewer's concern.

Reviewer #3: One of the interesting observations is that Fig. 2L shows brequinar induces p53 levels, if mildly, in both unselected and resistant SF188. However, there are no statistics given, nor any statement I saw the experiment was done in replicates or repeated multiple times.

Our response: We have included quantifications of the Western blot signals for γH2AX, p53 levels and MGMT levels (S2F, S2G and S2I-J Fig). In addition, more information on the methods and figures legends have been added to clarify the concerns raised by the reviewer. "*...(I) Western blot quantification by Image J of γ2AX in Fig 2K and additional experimental replicates. The results are represented for each experimental replicate. N=2. (J) Western blot quantification by Image J of p53 and MGMT in Fig 2K and additional experimental replicates. N=2 for p53, N=3 for MGMT.*"

References

1. Kofuji S, Hirayama A, Eberhardt AO, Kawaguchi R, Sugiura Y, Sampetrean O, et al. IMP dehydrogenase-2 drives aberrant nucleolar activity and promotes tumorigenesis in glioblastoma. Nat Cell Biol. 2019;21(8):1003-14. Epub 2019/08/03. doi: 10.1038/s41556-019-0363-9. PubMed PMID: 31371825; PubMed Central PMCID: PMCPMC6686884.
2. Aasland D, Gotzinger L, Hauck L, Berte N, Meyer J, Effenberger M, et al. Temozolomide Induces Senescence and Repression of DNA Repair Pathways in Glioblastoma Cells via Activation of ATR-CHK1, p21, and NF-kappaB. Cancer Res. 2019;79(1):99-113. Epub 2018/10/27. doi: 10.1158/0008-5472.CAN-18-1733. PubMed PMID: 30361254.
3. Barciszewska AM, Gurda D, Glodowicz P, Nowak S, Naskret-Barciszewska MZ. A New Epigenetic Mechanism of Temozolomide Action in Glioma Cells. PLoS One. 2015;10(8):e0136669. Epub 2015/08/27. doi: 10.1371/journal.pone.0136669. PubMed PMID: 26309255; PubMed Central PMCID: PMCPMC4550362.
4. Houtgraaf JH, Versmissen J, van der Giessen WJ. A concise review of DNA damage checkpoints and repair in mammalian cells. Cardiovasc Revasc Med.

2006;7(3):165-72. Epub 2006/09/02. doi: 10.1016/j.carrev.2006.02.002. PubMed PMID: 16945824.
5. Tolcher AW, Gerson SL, Denis L, Geyer C, Hammond LA, Patnaik A, et al. Marked inactivation of O6-alkylguanine-DNA alkyltransferase activity with protracted temozolomide schedules. Br J Cancer. 2003;88(7):1004-11. Epub 2003/04/03. doi: 10.1038/sj.bjc.6600827. PubMed PMID: 12671695; PubMed Central PMCID: PMCPMC2376384.
6. Castro GN, Cayado-Gutierrez N, Zoppino FC, Fanelli MA, Cuello-Carrion FD, Sottile M, et al. Effects of temozolomide (TMZ) on the expression and interaction of heat shock proteins (HSPs) and DNA repair proteins in human malignant glioma cells. Cell Stress Chaperones. 2015;20(2):253-65. Epub 2014/08/27. doi: 10.1007/s12192-014-0537-0. PubMed PMID: 25155585; PubMed Central PMCID: PMCPMC4326375.
7. Forsythe JA, Jiang BH, Iyer NV, Agani F, Leung SW, Koos RD, et al. Activation of vascular endothelial growth factor gene transcription by hypoxia-inducible factor 1. Mol Cell Biol. 1996;16(9):4604-13. Epub 1996/09/01. doi: 10.1128/mcb.16.9.4604. PubMed PMID: 8756616; PubMed Central PMCID: PMCPMC231459.
8. Sykes DB, Kfoury YS, Mercier FE, Wawer MJ, Law JM, Haynes MK, et al. Inhibition of Dihydroorotate Dehydrogenase Overcomes Differentiation Blockade in Acute Myeloid Leukemia. Cell. 2016;167(1):171-86 e15. Epub 2016/09/20. doi: 10.1016/j.cell.2016.08.057. PubMed PMID: 27641501; PubMed Central PMCID: PMCPMC7360335.
9. Lewis TA, Sykes DB, Law JM, Munoz B, Rustiguel JK, Nonato MC, et al. Development of ML390: A Human DHODH Inhibitor That Induces Differentiation in Acute Myeloid Leukemia. ACS Med Chem Lett. 2016;7(12):1112-7. Epub 2016/12/21. doi: 10.1021/acsmedchemlett.6b00316. PubMed PMID: 27994748; PubMed Central PMCID: PMCPMC5150668.
10. Fang J, Uchiumi T, Yagi M, Matsumoto S, Amamoto R, Takazaki S, et al. Dihydro-orotate dehydrogenase is physically associated with the respiratory complex and its loss leads to mitochondrial dysfunction. Biosci Rep. 2013;33(2):e00021. Epub 2012/12/12. doi: 10.1042/BSR20120097. PubMed PMID: 23216091; PubMed Central PMCID: PMCPMC3564035.
11. Lafita-Navarro MC, Perez-Castro L, Zacharias LG, Barnes S, DeBerardinis RJ, Conacci-Sorrell M. The transcription factors aryl hydrocarbon receptor and MYC cooperate in the regulation of cellular metabolism. J Biol Chem. 2020. Epub 2020/07/03. doi: 10.1074/jbc.AC120.014189. PubMed PMID: 32611766.
12. Echizenya S, Ishii Y, Kitazawa S, Tanaka T, Matsuda S, Watanabe E, et al. Discovery of a new pyrimidine synthesis inhibitor eradicating glioblastoma-initiating cells. Neuro Oncol. 2020;22(2):229-39. Epub 2019/09/10. doi: 10.1093/neuonc/noz170. PubMed PMID: 31499527.
13. Wang X, Yang K, Wu Q, Kim LJY, Morton AR, Gimple RC, et al. Targeting pyrimidine synthesis accentuates molecular therapy response in glioblastoma stem cells. Sci Transl Med. 2019;11(504). Epub 2019/08/09. doi: 10.1126/scitranslmed.aau4972. PubMed PMID: 31391321.
14. Chen X, Zhang M, Gan H, Wang H, Lee JH, Fang D, et al. A novel enhancer regulates MGMT expression and promotes temozolomide resistance in glioblastoma. Nat Commun. 2018;9(1):2949. Epub 2018/07/29. doi: 10.1038/s41467-018-05373-4. PubMed PMID: 30054476; PubMed Central PMCID: PMCPMC6063898.
15. Colaprico A, Silva TC, Olsen C, Garofano L, Cava C, Garolini D, et al. TCGAbiolinks: an R/Bioconductor package for integrative analysis of TCGA data. Nucleic Acids Res. 2016;44(8):e71. Epub 2015/12/26. doi: 10.1093/nar/gkv1507. PubMed PMID: 26704973; PubMed Central PMCID: PMCPMC4856967.

16. Estrada-Bernal A, Lawler SE, Nowicki MO, Ray Chaudhury A, Van Brocklyn JR. The role of sphingosine kinase-1 in EGFRvIII-regulated growth and survival of glioblastoma cells. J Neurooncol. 2011;102(3):353-66. Epub 2010/10/13. doi: 10.1007/s11060-010-0345-z. PubMed PMID: 20938717; PubMed Central PMCID: PMCPMC3085825.
17. Lafita-Navarro MC, Kim M, Borenstein-Auerbach N, Venkateswaran N, Hao YH, Ray R, et al. The aryl hydrocarbon receptor regulates nucleolar activity and protein synthesis in MYC-expressing cells. Genes Dev. 2018;32(19-20):1303-8. Epub 2018/09/27. doi: 10.1101/gad.313007.118. PubMed PMID: 30254109; PubMed Central PMCID: PMCPMC6169836.
18. Hao YH, Lafita-Navarro MC, Zacharias L, Borenstein-Auerbach N, Kim M, Barnes S, et al. Induction of LEF1 by MYC activates the WNT pathway and maintains cell proliferation. Cell Commun Signal. 2019;17(1):129. Epub 2019/10/19. doi: 10.1186/s12964-019-0444-1. PubMed PMID: 31623618; PubMed Central PMCID: PMCPMC6798382.
19. Venkateswaran N, Lafita-Navarro MC, Hao YH, Kilgore JA, Perez-Castro L, Braverman J, et al. MYC promotes tryptophan uptake and metabolism by the kynurenine pathway in colon cancer. Genes Dev. 2019;33(17-18):1236-51. Epub 2019/08/17. doi: 10.1101/gad.327056.119. PubMed PMID: 31416966; PubMed Central PMCID: PMCPMC6719621.
20. Xiao GY, Mohanakrishnan A, Schmid SL. Role for ERK1/2-dependent activation of FCHSD2 in cancer cell-selective regulation of clathrin-mediated endocytosis. Proc Natl Acad Sci U S A. 2018;115(41):E9570-E9. Epub 2018/09/27. doi: 10.1073/pnas.1810209115. PubMed PMID: 30249660; PubMed Central PMCID: PMCPMC6187133.
21. Bhave M, Mettlen M, Wang X, Schmid SL. Early and nonredundant functions of dynamin isoforms in clathrin-mediated endocytosis. Mol Biol Cell. 2020;31(18):2035-47. Epub 2020/06/25. doi: 10.1091/mbc.E20-06-0363. PubMed PMID: 32579424.
